# A novel coli myophage and antibiotics synergistically inhibit the growth of the uropathogenic *E. coli* strain CFT073 in stoichiometric niches

**DOI:** 10.1128/spectrum.00889-23

**Published:** 2023-09-21

**Authors:** Patiphan Khunti, Kittapart Chantakorn, Arishabhas Tantibhadrasapa, Htut Htut Htoo, Parameth Thiennimitr, Poochit Nonejuie, Vorrapon Chaikeeratisak

**Affiliations:** 1 Department of Biochemistry, Faculty of Science, Chulalongkorn University, Bangkok, Thailand; 2 Institute of Molecular Biosciences, Mahidol University, Nakhon Pathom, Thailand; 3 Department of Microbiology, Faculty of Medicine, Chiang Mai University, Chiang Mai, Thailand; 4 Research Center of Microbial Diversity and Sustainable Utilization, Chiang Mai University, Chiang Mai, Thailand; CCG-UNAM, Cuernavaca, Mexico

**Keywords:** Uropathogenic *E. coli*, bacteriophages, phage and antibiotic synergy, bacterial revival

## Abstract

Urinary tract infections are widespread bacterial infections affecting millions of people annually, with *Escherichia coli* being the most prevalent. Although phage therapy has recently gained interest as a promising alternative therapy for antibiotic-resistant bacteria, several studies have raised concerns regarding the evolution of phage resistance, making the therapy ineffective. In this study, we discover a novel coli myophage designated as Killian that targets *E. coli* strains, including the uropathogenic *E. coli* (UPEC) strain CFT073. It requires at least 20 minutes for 90% of its particles to adsorb to the host cells, undergoes subcellular activities for replication for 30 minutes, and eventually lyses the cells with a burst size of about 139 particles per cell. Additionally, Killian can withstand a wide variety of temperatures (4–50°C) and pHs (4 – 10). Genome analysis reveals that Killian’s genome consists of 169,905 base pairs with 35.5% GC content, encoding 276 open reading frames; of these, 209 are functionally annotated with no undesirable genes detected, highlighting its potential as an antibiotic alternative against UPEC. However, after an 8-hour phage treatment at high multiplicities of infection, bacterial density continuously increases, indicating an onset of bacterial growth revival. Thus, the combination study between the phage and three different antibiotics, including amikacin, ciprofloxacin, and piperacillin, was performed and showed that certain pairs of phage and antibiotics exhibited synergistic interactions in suppressing the bacterial growth revival. These findings suggest that Killian-antibiotic combinations are effective in inhibiting the growth of UPEC.

**IMPORTANCE:**

Phage therapy has recently been in the spotlight as a viable alternative therapy for bacterial infections. However, several studies have raised concerns about the emergence of phage resistance that occurs during treatment, making the therapy not much effective. Here, we present the discovery of a novel *E. coli* myophage that, by itself, can effectively kill the uropathogenic *E. coli*, but the emergence of bacterial growth revival was detected during the treatment. Phage and antibiotics are then combined to improve the efficiency of the phage in suppressing the bacterial re-growth. This research would pave the way for the future development of phage-antibiotic cocktails for the sustainable use of phages for therapeutic purposes.

## INTRODUCTION

Urinary tract infections (UTIs) are among the most common bacterial infections affecting millions of people each year (1). *Escherichia coli* is the predominant pathogen responsible for UTIs, along with other uropathogens such as *Proteus mirabilis*, *Klebsiella pneumoniae*, *Staphylococcus saphrophyticus*, *Staphylococcus aureus*, group B *Streptococcus*, and *Enterobacter* and *Enterococcus* species (2). Similar to other bacterial infections, treatment with antibiotics (3 – 5), along with antibiotic abuse, is fueling the rapid development of antibiotic resistance in uropathogens (6). Uropathogenic *E. coli* (UPEC) has been reported to be resistant to various classes of antibiotics, including ampicillin, cephalosporins, fluoroquinolones, aminoglycosides, and trimethoprim/sulfamethoxazole (7, 8). In European countries, nearly half of UPEC isolates are resistant to fluoroquinolones, while in Asia, including Thailand, this number reaches as high as 70% (9). Therefore, urgent action is needed to develop more effective treatments for bacterial infections in order to deal with the growing problem of antibiotic-resistant UPECs.

Phage therapy is becoming one of the most promising alternatives to antibiotic treatment due to its abundance, diversity, and exceptional specificity (10). Various studies have highlighted successful cases of phage therapy in diverse areas of treatment, including UTI (11, 12). For example, the isolated lytic phage VB_EcoS-Golestan, which showed favorable phage characteristics, including rapid adsorption, large burst size, and high stability across a wide range of pH and temperatures, exhibited effective growth inhibition activity against clinical isolates of multidrug-resistant (MDR) *E. coli*, highlighting its potential as an antibiotic alternative (13). An *in vivo* study also showed that the novel coliphage LM33_P1 reduced bacterial titers and increased the survival rates of mice in three infection models, including pneumonia, septicemia, and murine UTI (14). Phage effectiveness was also evaluated in clinical studies where a phage cocktail with broad activity against uropathogenic bacteria (the PYO phage) was used to treat patients with UTIs. The study found that the bacterial titers of 67% of the patients were reduced by between 1 and 5 logs with no bacteriophage-associated adverse effects after treatment (15).

Although phage therapy offers numerous advantages, the emergence of phage-resistant pathogens is inevitable (16). Studies have demonstrated that phage-resistant bacteria can rapidly emerge *in vitro*, and cross-resistance among different phages is common, which hinders the effective use of phage cocktails (17 – 19). Therefore, many researchers are investigating whether the use of genetically diverse phages displaying different mechanisms of pre-killing and phage-antibiotic combinations is a better option than using closely related phages or phage alone (19, 20). This is in part because, from an evolutionary perspective, the combination of two distinct selective pressures is more effective than either one alone and therefore more difficult to develop resistance against. For example, pathogens require different mutations to confer resistance against combination therapy, which is more difficult than resisting a single drug (21). As a result, numerous studies have demonstrated the successful use of phage-antibiotic combinations in curbing antibiotic-resistant pathogens (20, 22, 23). In particular, one study showed that the association between resistant phenotypes across clinical *E. coli* isolates was stronger for the same type of stressor (i.e., antibiotic-antibiotic and phage-phage) than a different type (i.e., antibiotic-phage). This finding suggests that the resistant phenotypes against antibiotics and phages are evolving independently, thus minimizing the chance of cross-resistance (24).

Here, we present a lytic *E. coli* myophage named Killian that has a high degree of host specificity for UPECs. Its genome consists of 169,905 base pairs with 35.5% GC and encodes 276 open reading frames (ORFs) with no undesired genes. Killian has an adsorption duration of approximately 20 minutes, a latent period of 30 minutes, and a burst size of around 139 particles per cell. In addition, Killian is resilient to a wide range of temperatures (4–50°C) and pHs (4 – 10). Although the phage Killian possesses desired characteristics for use as a therapeutic agent, when used as a single stressor, bacterial growth revival can be detected within 8 hours of treatment. We then perform the phage and antibiotic synergy (PAS) analysis between the phage and three different classes of antibiotics to investigate the phage-antibiotic pairs that result in a synergistic outcome. Lastly, we show that selected phage-antibiotic pairs, which lead to effective PAS, are effective in suppressing the growth of the bacteria.

## MATERIALS AND METHODS

### Bacterial strains and growth

All bacterial strains in this study were grown overnight in Luria-Bertani (LB) broth (Tryptone; Himedia, Cat. No. RM027, and Yeast extract; Himedia, Cat. No. RM014) at 37°C with rolling at 100 rpm.

### Enrichment of phage against UPECs

Wastewater was collected from 20 locations (designated as L1–L20). The phage enrichment process was carried out by using wastewater as a phage source. A 96-well plate containing 25 µL of *E. coli* CFT073 was mixed with 25 µL of 100-mM CaCl_2_, 750 µL of 10× LB broth, and 750 µL of wastewater. The plate was then incubated at 30°C for 48 hours. To remove the bacterium host, 100 µL of chloroform was then added to each well. The enrichment culture, 5 µL, was dropped onto the bacterial lawn of *E. coli* CFT073 and then incubated at 30°C for 16 hours to observe whether the enrichment culture would contain phages. The enrichment cultures containing putative phages were then kept at 4°C until use.

### Phage isolation and purification

To isolate and purify phages, the double-layer agar method was performed. Ten microliters of 10-fold diluted enrichment cultures from selected wastewater L17 (10^−2^ to 10^−10^ dilution) was prepared by mixing 50 µL of enrichment cultures with 450 µL of SM buffer, and each dilution was added into 200 µL of *E. coli* CFT073, followed by adding 5 mL of molten 0.35% LB top agar. The mixtures were poured onto 2% LB agar plates, and the plates were incubated at 30°C for 16 hours. After the incubation, the putative single plaques that were translucent were picked and re-suspended in 100 µL of SM buffer. This step was repeated at least four times until the plaque morphology of each purified phage was homogeneous.

After the fourth round, the web-lysis plates where near confluent lysis was observed were soaked with 5 mL of SM buffer and incubated at 30°C for 5 hours. The soaked SM buffer was taken and centrifuged at 9,000 rpm for 15 minutes. Then, the supernatant was filtered through the 0.45-micron filter in order to remove the remaining bacterial cells.

The purified phage lysate from the enrichment culture was named Killian. The titer of phage Killian was determined by spot test. During the spot test, each phage was serially 10-fold diluted using SM buffer (10^−3^ to 10^−10^ dilution), and 5 µL of the dilution of phage lysate Killian was dropped onto the bacterium lawn of *E. coli* CFT073. Plaques were visualized after incubation at 30°C for 16 hours, and then the PFU per milliliter was calculated.

### Conventional phage study: host range, adsorption assay, single-step growth curve, and phage stability

The host spectrum and efficiency of plating (EOP, average PFU on target bacteria divided by average PFU on host bacteria) of phage Killian were determined by a spot test to test the infectivity of each phage against various *E. coli* strains including UPECs. The EOP and standard deviation were computed. The average EOP value for Killian was categorized according to the efficiency of killing as “highly productive” (the ratio was 0.5 or more), “medium productive” (the ratio was between 0.1 and 0.5), “low productive” (the ratio was between 0.001 and 0.1), and “inefficient” (the ratio was below 0.001).

To evaluate phage adsorption, *E. coli* CFT073 was infected with Killian at a multiplicity of infection (MOI) of 0.01 and incubated at 30°C. At each time point of 0, 2.5, 5.0, 7.5, 10.0, 15.0, 20.0, 25.0, and 30.0 minutes, the sample was collected and passed through a 0.45-micron filter. Then, the double-layer agar method was performed, and after incubation at 30°C for 16 hours, PFU per milliliter was calculated in order to evaluate the adsorption time of Killian. For the one-step growth curve analysis, *E. coli* CFT073 was infected with Killian at an MOI of 0.01 and incubated at 30°C for 20 minutes. Unabsorbed phages were removed by centrifuging the sample at 12,000 × *g* for 5 minutes. After discarding the supernatant, the pellet was re-suspended in LB broth. The sample was harvested every 10 minutes for 90 minutes. Then, the cell suspension from each time point was used to perform the double-layer agar procedure. After the incubation at 30°C for 16 hours, PFU per milliliter was calculated in order to evaluate the phage burst size and the latent period of Killian.

For phage temperature tolerance, phage Killian was incubated at various temperatures such as 4, 20, 25, 30, 37, 40, 50, 60, and 70°C for 1 hour using a thermocycler. After the incubation, the infectivity of the samples was tested by spot test. For phage pH stability, phage Killian was mixed with SM buffer in a pH range of 1–10 and incubated at 30°C for 1 hour. After the incubation, the infectivity of the samples was tested by spot test. All experiments were carried out in triplicate.

### Killing profile

The log phase of *E. coli* CFT073 was prepared by subculturing the overnight culture at 1:100 dilution. The day culture was then grown until optical density (OD_600_) reached 0.4 by shaking at 250 rpm, 30°C. The culture of 100 µL of *E. coli* CFT073 was then added into 96-well plates and infected with Killian at various MOI as follows: 0.01, 0.1, 1.0, 10.0, and 100.0. The final volume in each well was adjusted to 200 µL using LB broth. All infected cultures were monitored by measuring the OD_600_ every 15 minutes for 24 hours. This experiment was carried out in triplicate.

### Transmission electron microscopy

Phage lysate of Killian was precipitated with 10% wt/vol polyethylene glycol and 1-M NaCl for 16 hours. The precipitated lysates were centrifuged at 9,000 rpm for 10 minutes, and then the pellets were re-suspended in SM buffer. The morphology of phages was visualized by negatively staining with 2% uranyl acetate on a carbon-coated grid using transmission electron microscopy (TEM) (HITACHI model HT7700).

### Lysogeny test

Bacterial colonies that grew in a double-layer agar plate at a high titer of phage Killian were selected and further purified in order to identify phage-resistant strains. These resistant isolates were then confirmed as *E. coli* by MALDI Biotyer and kept frozen at −80°C for later use. The isolated strains and the original strain of *E. coli* CFT073 were cross-streaked with a drop of high-titer phage lysate on top of the bacterial stripes to confirm the phage resistance. The plate was incubated at 30°C for 16 hours, and the result was recorded. To test whether the phage-resistant isolates were lysogenic, the phage-resistant isolates were exposed to UV for 10 seconds in order to activate the prophage. Then, the phage-resistant isolates were picked by a sterile toothpick and stabbed onto the fresh bacterium lawn of *E. coli* CFT073. The plates were incubated at 30°C for 16 hours. The presence of a clear zone after the incubation indicates the presence of phage released from the resistant isolates.

### Phage genomic DNA extraction

With the use of the phenol-chloroform-isoamyl alcohol technique, the DNA of the phage Killian was isolated, as described previously (25). In order to precipitate the phage particles, 10% wt/vol of polyethylene glycol and 1-M NaCl were combined with phage lysate that contained at least 10^9^ PFU/mL. The phage precipitant was centrifuged for 10 minutes at 8,500 rpm. The host DNA and RNA were then removed by re-suspending the phage precipitant in SM buffer and treating it with 10 U of DNase I and 0.1 mg/mL of RNase A. Twenty millimolar EDTA was used to suppress the DNase and RNase activities, and 0.5-mg/mL proteinase K and 0.5% SDS were used to break down the phage capsid. After the incubation at 55°C for 1 hour, phenol-chloroform-isoamyl alcohol (25:24:1) (Sigma Aldrich, Switzerland) was added to the mixture at equal volumes. The mixture was mixed gently before centrifuging at 15,000 × *g* for 10 minutes. The aqueous layer at the top was collected. The steps of phenol-chloroform-isoamyl alcohol addition and centrifugation were repeated before being treated with 1/10 vol of 3-M sodium acetate and 2 vol of cold absolute ethanol. The genomic DNA was centrifuged at 21,000 *× g* for 20 minutes after being allowed to precipitate at −20°C for 3 hours. After rinsing with 70% ethanol and centrifuging at 15,000 × *g* for 5 minutes, the DNA pellet was left to air-dry until all of the ethanol had evaporated. The phage DNA was dissolved in the Tris-EDTA (TE) buffer. The quantity and quality of the DNA were evaluated using a NanoDrop 2000 spectrophotometer.

### Whole-genome sequencing and bioinformatic analysis

Whole-genome sequencing of Killian was done by using MiSeq (Illumina); the data were obtained as a FASTQ file and then was assembled with SPAdes. The single contig of the Killian genome was annotated using DNA master version 5.23.6 (Glimmer version 3.02 and GeneMark HMM). The prediction of functional proteins was manually performed using viral genomes available in the National Center for Biotechnology Information (NCBI) database, PHASTER, and NCBI conserved domains. Killian genome map was created by using Artemis: DNAPlotter version 18.1.0. Pairwise intergenomic similarities between related phage genomes were performed by using Virus Intergenomic Distance Calculator (VIRIDIC) (26) in order to calculate intergenomic similarity for phage clustering. The genomes of the phages were obtained from GenBank, the accession numbers being as follows: OV876900.1, OV877085.1, OP072608.1, NC_055723.1, MZ753803.1, MZ234041.1, MZ234030.1, MZ234053.1, MZ234040.1, and MZ234039.1. For phylogenetic analysis of Killian and the most closely related phages, the proteomic tree was calculated using whole-genome-wide sequence similarities by tBLASTx using ViPTree software (27). Moreover, Taxonomy classification and phage life cycle were predicted by using PhageAI software (28).

### Minimum inhibitory concentration

The minimum inhibitory concentration (MIC) of antibiotics against a bacterial strain was determined using a microdilution method (29). Briefly, a bacterial subculture was prepared by diluting an overnight culture in LB broth to an OD_600_ of 0.2. Subsequently, antibiotics including amikacin (AMK), piperacillin (PIP), and ciprofloxacin (CIP) were diluted in a 96-well plate using twofold serial dilution, with concentrations ranging from 32.0 to 0.0625 µg/mL for amikacin and piperacillin, and from 1.0 to 0.002 µg/mL for ciprofloxacin. Then, 1 µL of the subculture was diluted in LB broth and added to each well so that the final concentration of bacteria in each well would be 2 × 10^5^ CFU/mL. The plate was incubated at 37°C overnight, and the well with the lowest concentration of antibiotics that exhibited no visible bacterial growth was considered the MIC.

### Phage-antibiotic interaction analysis via checkerboard assay

To prepare the subculture, 100 µL of an overnight culture was inoculated into 10 mL of LB broth and incubated at 37°C until an OD_600_ of 0.2 was reached. Phage lysate was subjected to 10-fold serial dilutions and then added to a final concentration ranging from 10^10^ to 10^4^ PFU/mL for the final MOI range from 10^2^ to 10^−4^. In a 96-well plate, the different dilutions of phage lysates were added from top to bottom, and different concentrations of antibiotics ranging from 1/16× MIC to 16× MIC were added from left to right. One hundred microliters of subculture was then added to each well of the 96-well plate so that the final bacterial concentration in each well was 10^8^ CFU/mL. The plate was then incubated at 37°C, and the OD_600_ was recorded every 15 minutes for a 24-hour period, with continuous shaking using a CLARIOstar plus instrument. The interaction type identification of each combination was analyzed using interaction plots based on three independent experiments, according to a previous study (30). Briefly, the percentage of reduction of the cells from both single and combined treatments was calculated as a relative growth reduction (%) by subtracting their endpoint measurements from the positive control. The relative growth reduction was calculated as (positive control OD − treatment OD) / positive control OD × 100. Interaction plots were used to identify the relationship between each individual condition, while two-way analysis of variance (SPSS statistics version 18.0) was applied separately to each interaction for statistical analysis.

### Phage-antibiotic interaction confirmation via cell viability count

To verify the interaction types of phage-antibiotic combinations, we examined the CFU of *E. coli* CFT073 after treating with the combination of phage at MOI of 1 and three antibiotics (1/16× MIC to 2× MIC) and compared with those that were treated with a single stressor alone. Initially, a subculture was prepared by inoculating 100 µL of an overnight culture into 10 mL of LB broth, which was then incubated at 37°C until the OD_600_ reached 0.2. Subsequently, 100 µL of the subculture was added to each well of a 96-well plate. The phage lysate was diluted with SM buffer and added to each well to achieve a final concentration of 10^8^ PFU/mL (MOI 1). Following that, amikacin, ciprofloxacin, and piperacillin were individually added to each well of the 96-well plate to achieve the desired concentration (1/16× MIC to 2× MIC). The plates were incubated at 37°C with shaking at 200 rpm for 24 hours. To determine cell viability, the cell suspensions were diluted and spotted onto LB agar. The diluted suspensions, ranging from 10^−1^ to 10^−9^, were spotted onto LB agar and incubated at 37°C overnight. The resulting colonies were counted and the log CFU per milliliter was calculated. Finally, the data of the bacterial growth control, antibiotics alone control, phage-alone control, and phage-antibiotic combination were compared. Data from at least three independent experiments were included in the statistical analysis using a two-tailed Student’s *t*-test.

### Determination of relative bacterial growth revival and the corresponding number of phage particles

To investigate the long-term effects of phage-antibiotic combinations, we assessed the relative revival of bacterial growth and the phage titers on days 1 and 5 of incubation according to a previous study (19). Briefly, desired phage-antibiotic combinations were prepared in the 96-well plates as mentioned above and incubated for 5 days. For sample collection at each time point, the cell suspension was aliquoted and diluted with 0.85% normal saline. The diluted suspensions, ranging from 10^−1^ to 10^−9^, were spotted onto LB agar and incubated at 37°C overnight prior to colony counting. The relative bacterial growth revival was calculated by converting CFU per milliliter to log CFU per milliliter and normalizing the data by dividing the log CFU per milliliter of the treated samples (phage-alone control and phage-antibiotic combination) by those of the bacterial growth control. Finally, the phage-antibiotic combination was compared to the phage-alone control to assess the relative effect of the combination. Simultaneously, to determine the remaining phage titers, the cell suspension from each time point was aliquoted and filtered through a 0.45-µM filter. The phage lysate was then diluted with SM buffer. The diluted phage lysate, ranging from 10^−1^ to 10^−7^, was spotted on LB agar overlaid with bacterial culture. The plates were incubated at 37°C overnight, and the plaques were counted and converted into log PFU per milliliter to quantify the remaining phages. Data from at least three independent experiments were included into the statistical analysis. The linear regression plot was generated via Seaborn version 0.11.2 (31) in Python. Statistical analysis of relative bacterial growth revival between the two conditions (phage alone vs. phage-antibiotic) of each day was calculated using two-tailed Student’s *t*-test.

## RESULTS

### Morphological and biological properties of phage Killian

**FIG 1:**
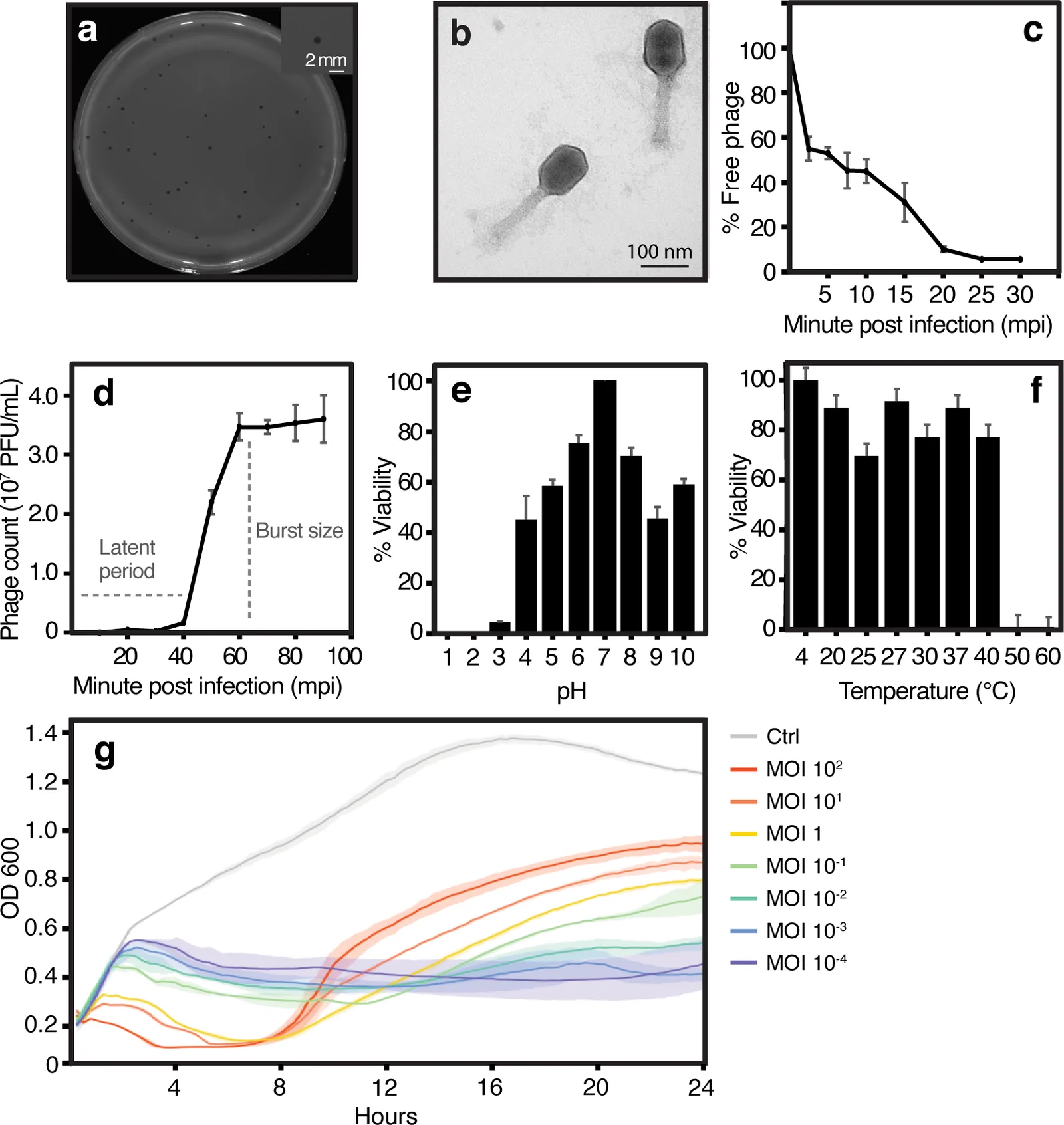
Morphological and biological properties of phage Killian. (a) Morphology of plaques produced by phage Killian on the lawn of UPEC strain CFT073. The panel on the upper right displays an individual plaque of phage Killian. Scale bar equals 2 mm. (b) Morphology of purified phage Killian as observed under a transmission electron microscope (TEM). Scale bar equals 100 nm. (c through g) Biological studies of phage Killian. (c) Adsorption assay. (d) One-step growth curve. (**e and **f) pH and thermal stability. (g) The killing profile of phage Killian against UPEC strain CFT073 at various MOIs (10^−4^ to 10^2^) at 37°C for 24 hours. (c through g) Experiments were performed in at least triplicates, and data were represented as the mean ± standard deviation.

Through phage enrichment using UPEC strain CFT073 as a bacterial host, a newly isolated phage, designated Killian, was isolated from sewage samples in Thailand. Killian was capable of lysing the host and producing plaques with a diameter of less than 1 mm, clear centers, and sharp edges (Fig. 1a). Rather than the parental host, UPEC strain CFT073, Killian exhibited a relatively broad spectrum of killing toward different *E. coli* strains (75%), as shown in Table 1. As determined by positive plaque formation and EOP, Killian was considered highly productive against the lab, UPECs, and probiotic strains, accounting for 50% of the tested strains. Despite the inefficient infectivity of Killian against clinical isolates, it could lyse half of the bacteria tested, indicating the therapeutic potential in a clinical setting (Table 1).

**TABLE 1:** Host spectrum of the phage Killian and efficiency of plating (EOP)^*a*^

Types of hosts		Source	Plaque formation	EOP	Class of EOP
Lab strains	*E. coli* ATCC25922	American Type Culture Collection	+	2.05	Highly productive
	*E. coli* MC4100	American Type Culture Collection	+	2.15	Highly productive
UPECs	*E. coli* CFT073	American Type Culture Collection	+	0.92	Highly productive
	*E. coli* UTI89	Derived from a cystitis patient 32 (33)	+	≤0.001	Inefficient
	*E. coli* ABU83972	Derived from a patient with asymptomatic bacteriuria 34 (35)	+	1.38	Highly productive
Probiotic strain	*E. coli* Nissle 1917	Wild-type strain (O6:K5:H1) 36 (37)	+	2.05	Highly productive
Clinically isolated UPECs	PK1036 (AT1)	Clinical strain isolated from the urine of cystitis patients hospitalized at Maharaj Nakorn Chiang Mai Hospital, Chiang Mai University, Thailand	−	–	–
	PK1037 (AT2)		+	≤0.001	Inefficient
	PK1038 (AT3)		+	≤0.001	Inefficient
	PK1039 (AT4)		−	–	–
	PK1040 (AT5)		−	–	–
	PK1041 (AT6)		+	≤0.001	Inefficient

^*a*^ The host range of phage Killian was tested against a variety of *E. coli* strains, including lab strains*,* and clinical UPECs. The positive plaque formation (+) is evaluated based on the presence of a clear zone of bacterial lawn where the phage was added. EOP (mean of PFU on the target bacteria divided by mean of PFU on the parental bacteria) was computed from three independent replicates. According to the efficiency of phage reproductivity, the average EOP value for a specific phage and bacterium combination was categorized as “highly productive” when the ratio was 0.5 or more, “medium productive” when the ratio was between 0.1and 0.5, “low productive” when the ratio was between 0.001 and 0.1, and “inefficient” when the ratio was below 0.001. “–” indicates that no plaques were formed.

As observed by TEM and classified by the International Committee on Taxonomy of Viruses guidelines, Killian was classified as a myophage as it possessed a prolate or an elongated icosahedral capsid with an estimated height and width of 102.4 ± 2.0 nm and 81.2 ± 1.3 nm, respectively, and a tail that was 168.0 ± 2.1 nm long (Fig. 1b, *n* = 5). Killian required at least 20 minutes for 90% of the phage particles to adhere to the host cells. During the latent period in the lytic cycle, it performed subcellular activities and propagated inside the host cells for roughly 40 minutes before releasing its offspring with a burst size of 139 particles per cell (Fig. 1c and d). Killian was quite stable over a wide range in both pH and temperature. It retained 40%–100% infectivity at pH 4–10 and from temperatures ranging from 4 to 40°C. The phage became fully inactivated at pH less than 3 and temperatures above 50°C (Fig. 1e and f).

Killian was highly infective against UPEC strain CFT073 as it strongly suppressed bacterial growth at various MOIs (Fig. 1g; Table 1). Interestingly, we found that when bacteria were treated with MOI of 1–100, although the bacterial growth was rapidly suppressed at early time points, the revival of bacteria was detected as early as 8 hours after incubation. Bacteria that were incubated with MOI of 10^−1^ of phage resulted in revival growth at 12 hours, while treating bacteria with lower concentrations of phage (MOI 10^−2^ to 10^−4^) resulted in more sustainable growth inhibition throughout the course of the 24-hour incubation. These results suggested that phage Killian is effective in inhibiting the growth of bacteria at low doses, but the effectiveness is less sustained at higher doses.

### Genome features and phylogenetic tree analysis of phage Killian

**FIG 2:**
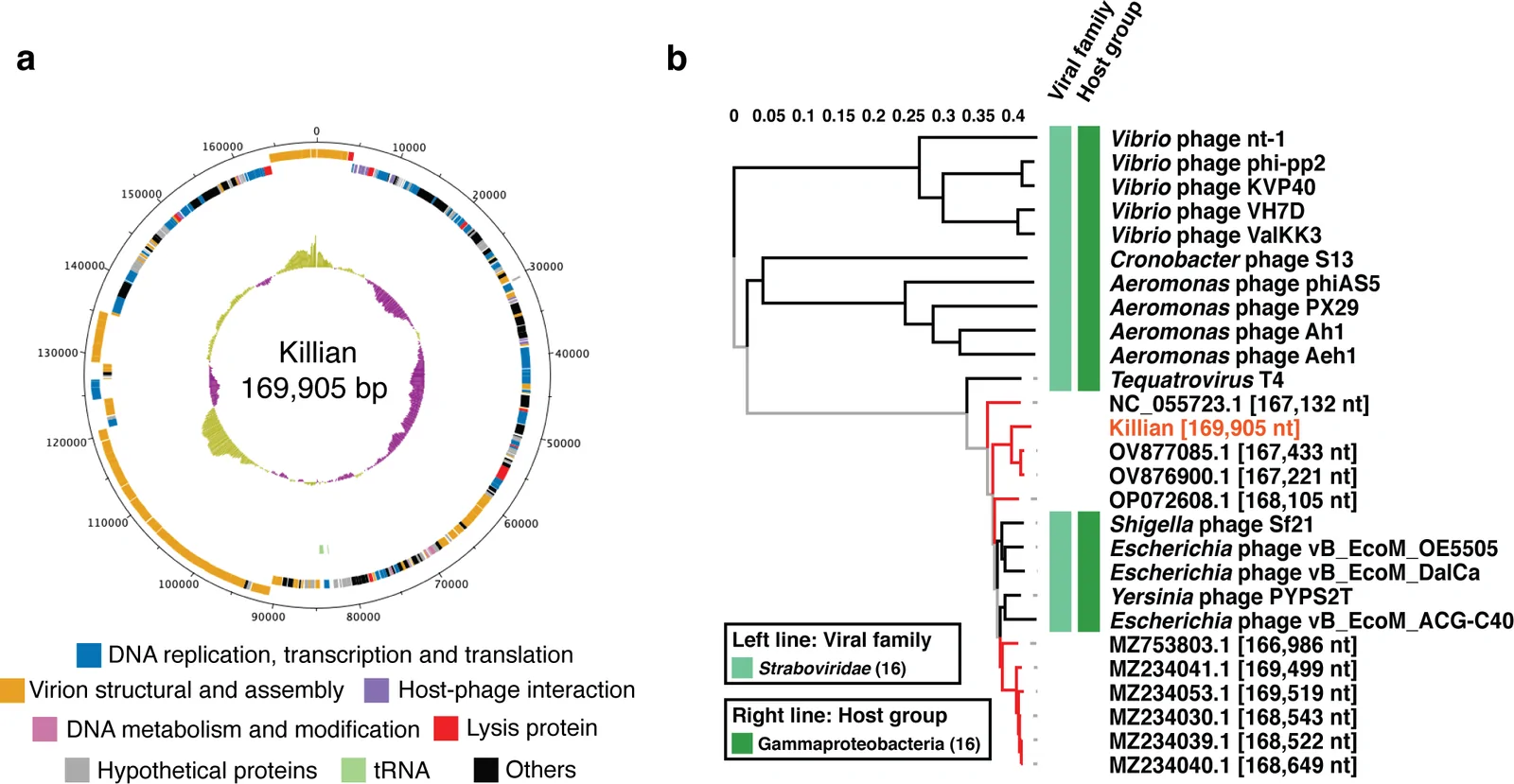
Phage Killian is a T4-like bacteriophage and harbors a relatively large genome size. (a) Genomic map of phage Killian. Killian’s ORFs are functionally annotated and divided into different groups as indicated by colors as follows: blue, DNA replication, transcription, and translation; pink, DNA metabolism and modification; orange, virion structural and assembly; purple, host-phage interaction; red, lysis protein; gray, hypothetical protein; green, tRNA; and black, others. An inner circular plot presents the GC content across the genome (yellow means above average, and purple means below average). The map was generated using Artemis: DNAPlotter version 18.1.0. (b) The proteomic tree of phage Killian with other closely related phages. The tree was constructed by ViPTree software.

Through whole-genome sequencing using the Illumina platform and assembly by SPAdes with coverage over 3,000×, a complete double-stranded DNA genome of Killian was obtained. The genome was 169,905 base pairs long, had a GC content of 35.4%, and encoded 276 ORFs (Fig. 2a; Table 2; accession number OQ446694 and BioProject number PRJNA957703). Of these, 209 ORFs were designated as functionally predicted genes with significant hits for functional annotation (Blastx search E-values less than 10^−4^). The remaining 67 ORFs were classified as hypothetical proteins. We also confirmed the functional annotation through protein structure databases (Table S1). The functionally predicted ORFs were categorized into 51 genes related to DNA replication, transcription, and translation; 7 genes related to DNA metabolism and modification; 81 genes related to virion structure and assembly; 9 genes for host-phage interaction proteins; 9 genes related to host cell lysis proteins; and 45 genes for other phage-related proteins. The genome was found to harbor seven tRNA-encoding genes. Additionally, through extensive annotation through various databases and software, including NCBI Blast, NCBI Conserved Domain, PHASTER, and Phage AI, the analyses revealed that the genome did not contain any toxins, drug-resistant genes, or lysogenic-associated genes. Specifically, PhageAI software demonstrated that Killian was a virulent phage with a 96.04% confidence level of prediction, rendering it proper for therapeutic use. This prediction was further validated by a lysogeny test to examine if the phage is temperate and capable of integrating its genome into the host. We first isolated 10 Killian-resistant isolates, confirmed the bacterial species by MALDI Biotyper, and induced the lysogen, if any, from these isolates. The result showed that, after the activation, none of the resistant strains produced plaques (Fig. S1), confirming the genome analysis that Killian is a lytic phage and does not enter the lysogenic cycle.

**TABLE 2:** List of selected annotated proteins from ORFs in the genome of phage Killian^*a*^

Group	ORF	Predicted function	Direction	Start	Stop	Size (n)	Sequence similarity	Accession no.	Database
DNA replication, transcription, and translation	ORF14	DNA topoisomerase	−	7,983	9,311	1,329	DNA topoisomerase *Shigella* phage ESh26	URY13538	NCBI
	ORF73	DNA polymerase	−	38,949	41,645	2,697	DNA polymerase (*Escherichia* phage UGKSEcP2)	CAH1615347.1	NCBI
	ORF75	DNA polymerase clamp loader subunit A	−	42,094	42,657	564	DNA polymerase clamp loader subunit A (*Escherichia phage* vB_EcoM-CHD94UKE2)	QZI80988	NCBI
	ORF249	DNA helicase	−	149,309	149,629	321	PHAGE_Shigel_SHFML_26_NC_031011: DNA helicase	PP_00245	PHASTER
DNA metabolism and modification	ORF4	DNA-binding transcriptional regulator	−	4,483	4,755	273	PHAGE_Escher_CF2_NC_041919: DNA-binding transcriptional regulator, phage(gi100278)	PP_00004	PHASTER
	ORF78	RNA polymerase-binding protein	−	44,410	44,799	390	RNA polymerase-binding protein (*Escherichia* phage vB_EcoM_SA20RB)	UIU27997	NCBI
	ORF89	RNA polymerase sigma factor	−	51,115	51,672	558	RNA polymerase sigma factor (*Escherichia* phage vB_EcoM_112)	YP_009030673	NCBI
	ORF271	Late promoter transcription accessory protein	−	162,526	162,864	339	Late promoter transcription accessory protein (*Escherichia* phage T4)	NP_049857.1	NCBI
Virion structural and assembly	ORF1	Tail fiber protein	+	30	2,978	2,949	Tail fiber protein (*Escherichia* phage vB_EcoM_Shinka)	QXV73019	NCBI
	ORF127	Major tail protein	−	70,253	70,441	189	PHAGE_Escher_CF2_NC_041919: major tail protein	PP_00130	PHASTER
	ORF203	Major capsid protein	+	117,724	119,289	1,566	Major capsid protein (*Shigella* phage ESh30)	URY14669.1	NCBI
	ORF217	Base plate protein	−	128,243	128,869	627	T4 bacteriophage base plate protein (*Escherichia* phage slur07)	YP_009197268.1	NCBI
Host-phage interaction	ORF6	Antirestriction nuclease	−	4,917	5,195	279	Antirestriction nuclease (*Shigella* phage ESh25)	URY13403	NCBI
	ORF16	Nuclear disruption protein	−	9,695	10,150	456	Nuclear disruption protein (*Escherichia* phage ime09)	WP_016059142	NCBI
	ORF62	Spackle periplasmic protein	−	32,242	32,535	294	Spackle periplasmic protein (*Shigella* phage SHFML-26)	YP_009279032.1	NCBI
	ORF70	Immunity to superinfection membrane protein	−	37,860	38,111	252	Immunity to superinfection membrane protein (*Escherichia* phage vB_EcoM_SYGD1)	QUD16027	NCBI
Lysis protein	ORF3	Holin protein	+	3,826	4,482	657	Holin protein (*Escherichia* phage vB_EcoM_SP1)	QLF80826	NCBI
	ORF11	Lysozyme	−	6,733	7,368	636	PHAGE_Shigel_Sf24_NC_042078: lysozyme	PP_00013	PHASTER
	ORF82	Endolysin	−	46,919	47,182	264	PHAGE_Escher_slur04_NC_042130: endolysin, phage(gi100033)	PP_00083	PHASTER
	ORF252	Putative spanin inner membrane subunit	−	150,149	150,502	354	Putative spanin inner membrane subunit (*Escherichia* phage 132)	QWY90593	NCBI

^*a*^ ORFs with the predicted functions were determined by their significant hit (E-value <10^−4^) against the genome databases (see accession number OQ446694 for details).

To investigate whether the phage is novel, we chose closely related phages whose genomes are highly similar to the genome of phage Killian, based on BLASTn searches, and then used VIRIDIC to assess intergenomic similarity. The result revealed that the genome sequence of Killian was highly similar to *Escherichia* phages OV876900.1 and OV877085.1 at 95.0 and 94.7%, respectively (Fig. S2). However, due to the intergenomic similarity value between Killian and these closely related phages being less than 96% (26), Killian was thus clustered into different species from these related phages, indicating the novelty of the phage. To reveal how Killian is phylogenetically clustered, a proteomic tree was then constructed from the genomes of closely related phages as obtained from the NCBI database, based on the similarities of the whole-genome sequences with Killian, and other selected phages as available in the ViPTree software (Fig. 2b). The tree confirmed the close relationship of Killian with OV876900.1 and OV877085.1 as they were clustered in the same branch and further demonstrated that Killian was one of the T4-like viruses as it was clustered with other *E. coli* phages, including phage T4 (Fig. 2b).

### Unique combinations of Killian and antibiotics revealed PAS

**FIG 3:**
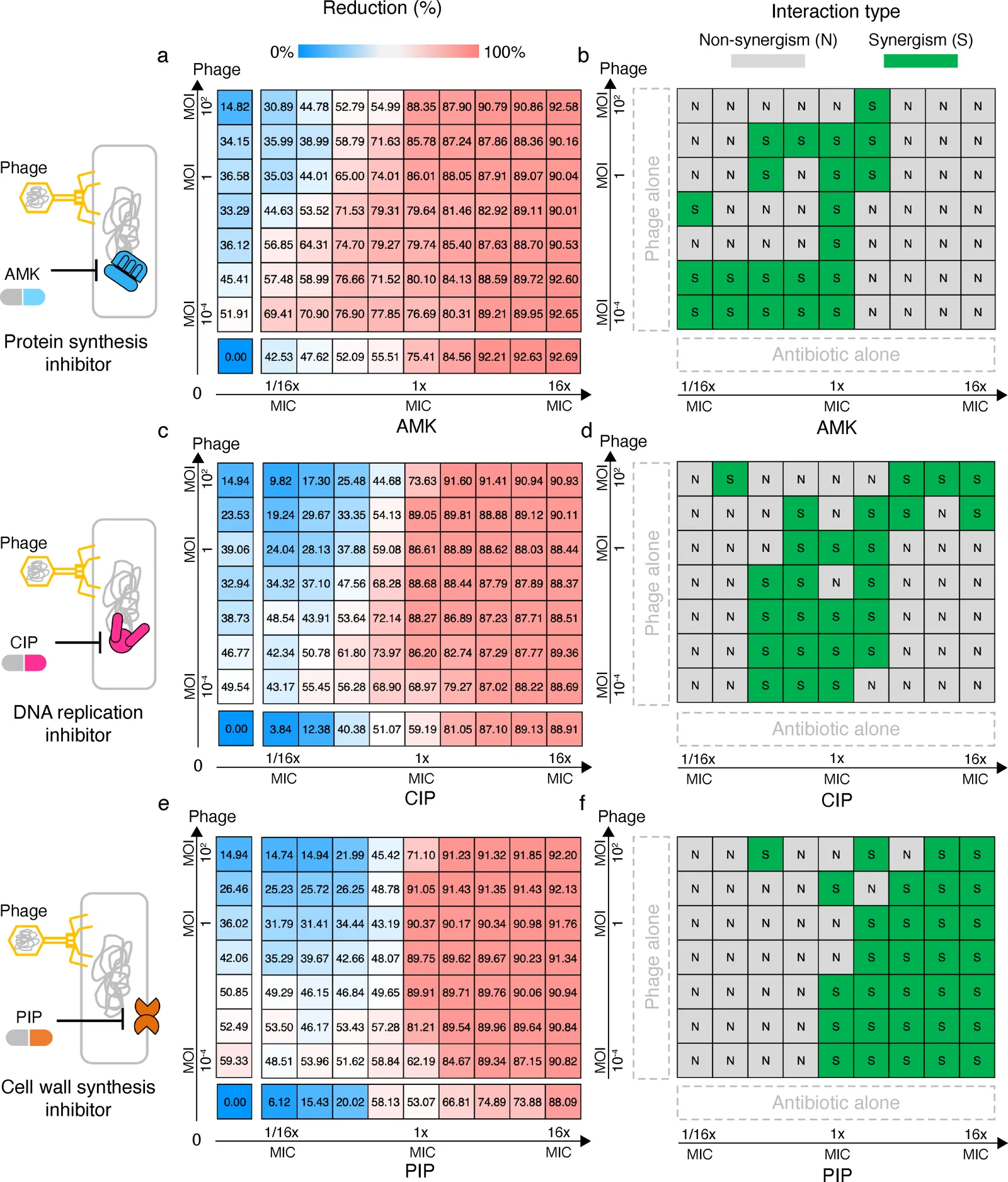
Killian-antibiotic combinations by two-dimensional checkerboard analysis showed various interaction types, depending on stoichiometry. The average percentage of growth reduction of UPEC strain CFT073 after 24-hour treatment with phage Killian (MOI 10^−4^ to 10^2^) in combination with (a) AMK, (c) CIP, (e) PIP (1/16× MIC to 16× MIC). The interaction types (non-synergism, N; and synergism, S) of Killian in combination with (b) AMK, (d) CIP, and (f) PIP against UPEC strain CFT073 at various conditions. Interaction types are determined by comparing each condition with those of the single stressors. Two-way ANOVA was applied to each interaction for statistical analysis.

Since the bacterial regrowth was detected within 8 hours of phage treatment (Fig. 1g), especially at high doses of Killian, we asked if the use of a phage-antibiotic combination could provide a complementary solution to using phage as an effective therapeutic agent. A previous study shed light on the combination of phages and antibiotics that results in greater bacterial growth inhibition, which varies greatly, depending on various factors, especially the types and concentrations of the antibiotics in the combination (30). Thus, we first investigated which antibiotics, and at what concentrations, would effectively reduce the growth of the bacteria: UPEC strain CFT073. The selected antibiotics in this study included AMK (protein synthesis inhibitor), CIP (DNA replication inhibitor), and piperacillin or PIP (cell wall synthesis inhibitor). We first determined the MIC of each antibiotic against the bacteria and found that the MICs were 24 µg/mL for AMK, 0.023 µg/mL for CIP, and 2 µg/mL for PIP. By performing a two-dimensional checkerboard analysis at 24 hours of phage (MOI 10^−4^ to 10^2^) with these three different antibiotics (1/16× MIC to 16× MIC), we found that each antibiotic resulted in a different growth reduction pattern when used together with the phage (Fig. 3). For AMK and CIP, when the antibiotic concentration used in the combination was below their MIC level, the addition of phage at almost all MOI greatly reduced the bacterial growth when compared to a single stressor alone (Fig. 3a and c). This result suggested a possible synergistic interaction between the phage and the antibiotics at levels below the MIC level of AMK and CIP. On the contrary, when the PIP concentration was below its MIC, the combination of PIP and phage resulted in a similar or even decrease in the growth reduction percentage when compared to a single stressor alone (Fig. 3e). However, we found that, at the MIC or above, the combination resulted in a higher growth reduction rate, which might indicate the synergistic interaction at higher concentrations of PIP.

In order to swiftly reveal which combinations give rise to possible PAS outcomes, we evaluated the growth of the bacteria in the checkerboard at 24 hours and interpreted the interaction type according to criteria used in a previous study (30) (Fig. S3); see Materials and Methods for more details). Similar to the growth reduction data, the combination that greatly reduced the growth of bacteria when compared to the single stressor alone generally resulted in a synergistic interaction, while the combination that resulted in lesser inhibition or did not differ significantly from using a single stressor alone, were designated as non-synergism (Fig. 3b, d and f). Altogether, the checkerboard assay showed that different antibiotics in combination with Killian resulted in different niche combinations where desired PAS outcomes were observed.

### Cell viability count confirmed synergistic effect of PAS pair candidates

**FIG 4:**
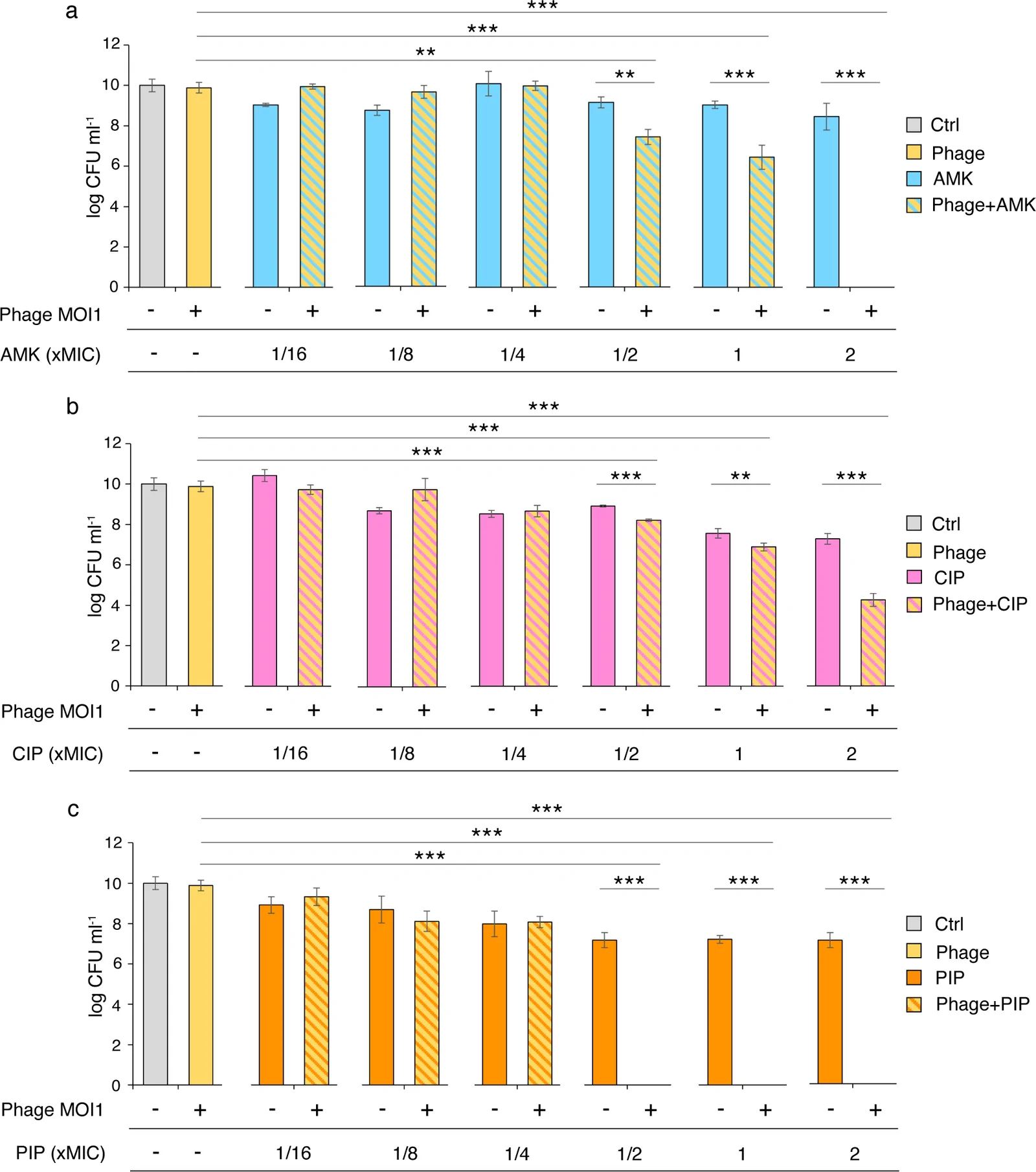
Combinations of Killian (MOI 1) and antibiotics (1/2× to 2× MIC) synergistically reduced the bacterial cell viability count. Number of log CFU per milliliter of *E. coli* CFT073 in (a) phage + AMK , (b) phage + CIP, and (c) phage + PIP combination experiments at 24 hours. Values and standard deviations are from at least three independent experiments, per condition. **P < 0.01, ***P < 0.001; two-tailed Student’s *t*-test.

Although the checkerboard assay could be used to swiftly screen through a vast array of phage-antibiotic interactions and reveal possible PAS pair candidates, the method’s reliance on the cell turbidity measurement might hamper the accuracy of the interaction interpretation. This was also observed in our study in which some of the phage-antibiotic interactions as identified by the checkerboard assay are in contradiction with those of previous studies (38 – 41). In particular, for PIP, which was previously reported to synergistically work with *E. coli* phage (23), we found that a certain number of interactions were defined as non-synergistic in our study, especially in those that contain PIP at below 1× MIC level (Fig. 3f). Thus, in order to confirm the interaction type of phage-antibiotic pairs, cell viability count of the bacteria treated with phage (MOI 1) and three antibiotics (1/16× MIC to 2× MIC) was performed. Similar but not identical to the checkerboard assay, the result showed that the combination of phage Killian at MOI of 1 with AMK, CIP, or PIP at the 1/2 MIC level and above significantly reduced the number of bacteria when compared to a single stressor alone (Fig. 4). However, in the condition in which the concentration of the antibiotics is below 1/2× MIC, the combination did not significantly alter the number of bacterial counts. Overall, the results suggest that the antibiotics synergize with the phage in inhibiting the growth of the bacteria in a concentration-dependent manner.

### Effects of PAS pairs on bacterial growth revival and remaining pool of phages

**FIG 5:**
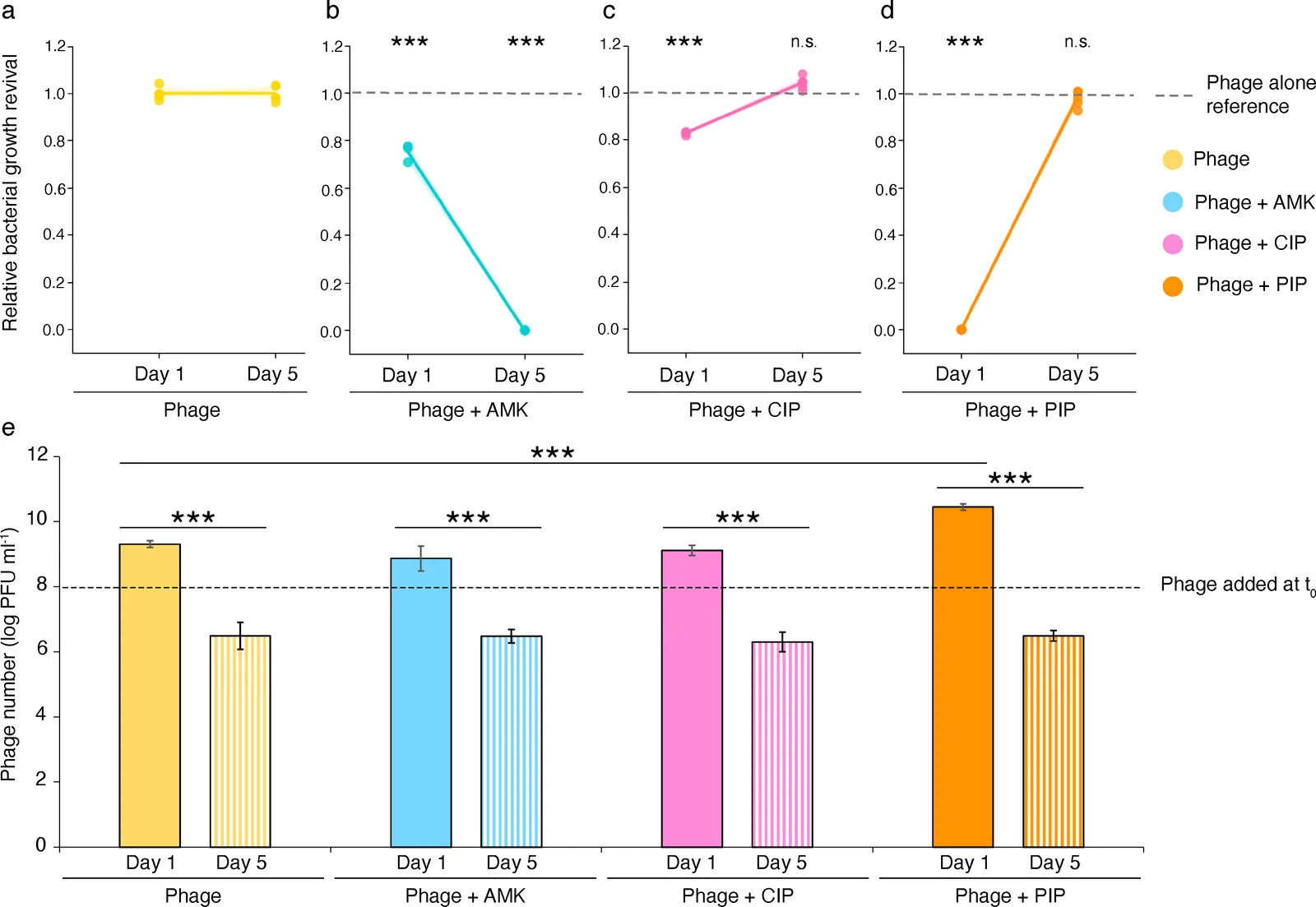
Effects of Killian (MOI 1) and antibiotics (1/2× MIC) combinations on bacterial growth revival and number of phages. Relative growth revival of *E. coli* CFT073 treated with (a) phage alone as a baseline reference, (b) phage + AMK, (c) phage + CIP, and (d) phage + PIP for 1 and 5 days. Shaded areas indicate 95% confidence. (e) Corresponding number of log PFU per milliliter of Killian phage from each condition. Values and standard deviations are from at least three independent experiments, per condition. ***P < 0.001; two-tailed Student’s *t*-test. n.s., not statistically significant.

While the cell viability determination at 24 hours supports the use of phage-antibiotic combinations, investigation of bacterial growth revival beyond the initial 24 hours would be beneficial for the long-term use of these combinations. Thus, the relative bacterial growth revival of UPEC strain CFT073 treated with selected PAS pairs (MOI 1 of phage + 1/2 MIC of antibiotics) that showed synergistic effect (Fig. 4) was further investigated at day 5 to provide additional data regarding the long-term growth inhibition effect of the pairs when compared to using phage alone. Interestingly, we found that different antibiotics, albeit their similar effect on the growth suppression at day 1, resulted in different remaining cell viability at day 5 (Fig. 5a through d). In particular, phage + AMK resulted in marginally lower growth revival at day 1; however, its synergistic effect was exacerbated at day 5 as there was no cell viability detected (Fig. 5b). On the contrary, while cell viability was undetectable at day 1 under phage + PIP treatment, bacterial growth revival at day 5 was indistinguishable from those treated with the phage alone (Fig. 5a and d). Similarly, but to a lesser degree, phage + CIP significantly reduced the bacterial growth revival at day 1; however, at day 5, the countable bacterial colony was at the same level as those treated with phage alone (Fig. 5a and c). These data suggested that the long-term synergistic effect of phage-antibiotics pairs can be diverse, depending on the types of antibiotics and time points evaluated.

One of the proposed major advantages of phage therapy, unlike chemotherapy, is an auto-dosing characteristic of the phage due to its ability to reproduce in the bacterial host (42). The fact that different antibiotics in the PAS pairs exhibited distinct effects on bacterial growth revival urged us to investigate whether the pairs contribute to any changes in the number of phages in the combination or not. As expected, we found that the concentration of phage in all conditions tested (phage alone, phage + AMK, phage + CIP, and phage + PIP) at day 1 is higher than the number of phages added at the beginning of the experiment (10^8^ PFU/mL). Interestingly, phage concentration in the phage + PIP treatment was higher than those of phage alone at day 1 (Fig. 5E). This finding is in agreement with the previous study, which also showed that the antibiotic that induced cell filamentation and weakened cell wall integrity (i.e., β-lactam) possibly increased the efficiency of the phage reproduction process and hence resulting in higher phage titer (23, 43). Also, the higher number of the phage in phage + PIP coincided with the undetectable bacterial cell count at the same time point (Fig. 5D and E), suggesting the relationship between host cell death and higher phage titer. We further found that, although the number of phages at day 1 was higher than the level added at the beginning of the experiment, the phage level at day 5 in all conditions was indifferent and was significantly lower than those of day 1 (Fig. 5E), suggesting that the effect of phage-antibiotics tested in this study do not drastically affect the number of phages in the combination beyond 1-d treatment.

## DISCUSSION

Phage therapy has recently attracted interest in the scientific community due to the promising outcomes of treatments as demonstrated by several research studies (44 – 46). Monophage therapy, a regime in which a single phage is used, provides exceptionally high specificity toward the target pathogens (47). However, long-term treatment with monophage therapy may result in the rapid development of bacterial resistance against the phage (48), hampering the treatment effectiveness. Here, we discover the myophage Killian that specifically targets uropathogenic *E. coli*, which exhibits the characteristics that are proper for biocontrol and therapeutic use. Even though Killian is highly effective in suppressing the bacterial growth, phage resistance can be developed after 8 hours of the treatment when Killian is used alone, indicating the limitation of monophage therapy when using phage Killian.

Killian is considered a novel T4-like myophage, which appears phylogenetically most closely related to *Escherichia* phage UGKSEcP2 (OV877085.1). Killian harbors a relatively large genome, in which it encodes numerous proteins (Table 2; Table S1) involved in DNA replication and metabolism, suggesting that the DNA metabolism is largely independent of the host DNA replication machinery. The proteins involved in DNA replication include DNA polymerase (ORF073), DNA polymerase clamp loader subunit A (ORF075), DNA helicase (ORF026, ORF210, ORF212, and ORF249), DNA ligase (ORF228), DNA recombinase (ORF067), DNA topoisomerase (ORF014), and DNA polymerase accessory proteins (ORF076 and ORF077). Moreover, due to the presence of various transcription modulators in the genome, such as transcriptional regulators and repressors (ORF004, ORF027, ORF036, ORF037, ORF051, ORF052, ORF074, ORF235, and ORF253), RNA polymerase-binding protein (ORF078), RNA polymerase sigma factor (ORF089), and late promoter transcription accessory protein (ORF271), phage Killian possibly interferes with the host transcriptional apparatus and takes over it for its own processes of gene expression and regulation. Since phage genomes have been considered as an untapped resource of antimicrobial discovery (49), we found nearly a complete set of lysis-related proteins that target the bacterial cell wall and cell membrane during phage egress, in which they would be developed for therapeutic use. The proteins necessary for host cell lysis include holin (ORF003), endolysin (ORF082, ORF100, ORF148, and ORF273), transglycosylase (ORF138), and spanin (ORF252). Other enzymes targeting the bacterial cell wall are also present in the genome, such as lysozyme (ORF011) and cell wall hydrolase (ORF038), further providing alternative therapeutics through the use of phage-derived antimicrobials. To investigate their antibacterial properties, further experiments to examine whether these phage proteins are bactericidal would be required.

It is well noted that, despite the great potential of phage therapy in curbing down the pathogen, phage-resistance emergence is unavoidable (16, 25). In this study, we found that bacterial growth declined rapidly at early incubation, followed by reemergence within 8 hours at MOIs ranging from 1 to 100, but none was detected at lower concentrations (MOI 10^−2^ to 10^−4^). Similar to a previous study of T4 phage (50), it is possible that a higher phage MOI could induce an early first lysis turbidity decline followed by an increase in cell density. Although there is no direct evidence to support the correlation between the use of high MOI and the early emergence of bacterial resistance against phage, some studies suggest that bacteria can mutate under higher selective pressure (32). Also, the relationship between phage number and the emergence of phage-resistant bacteria is complex and influenced by multiple factors (34). Therefore, another limitation worth noting in this study is that other factors, such as bacteria culture density and the environment, which can also contribute to bacterial resistance, are not yet fully investigated.

Due to the inevitable fate of phage-resistant mutant emergence, various studies have focused on using phage-antibiotic combinations, and their synergistic effects are commonly desired for therapeutic purposes (36). Although we found that all antibiotics tested exhibited PAS effects in combination with Killian, similar to a previous study of coliphage (23), conflicting interaction types of phage-antibiotic pairs between checkerboard and cell viability count are worth mentioning here. In particular, the combination of 1/2 and 1× MIC of PIP with Killian was identified as non-synergistic in the checkerboard (Fig. 3f) but significantly reduced the cell viability count (Fig. 4c). This contradictory finding might be due to the fact that the checkerboard assay monitored the growth of the bacteria via an OD_600_ reading, which correlates with the density of the bacterial culture, regardless of the size of the individual cell. Since it is well known that β-lactam-treated cells are much longer than the untreated cells (29, 51, 52), the OD_600_ reading could not be accurately converted to cell viability count. Thus, cell viability count evaluation in each condition is crucial in determining the phage-antibiotic interaction, thereby improving the overall accuracy of defining the type of phage-antibiotic interaction. It is also interesting to note that some of the findings here are also in contradiction with common perceptions toward certain phage-antibiotic interactions. In particular, while several studies have demonstrated that aminoglycosides and other protein translation inhibitors are antagonistic to the action of the phages due to the inhibition of the synthesis proteins (41, 53), here we found that the aminoglycoside AMK synergistically inhibited the growth of the bacteria, with phage, in a concentration-dependent manner (Fig. 4a). Similarly, in a previous study, Malik *et al* showed that *Escherichia* phages were able to reduce the MIC of AMK, resulting in a synergistic effect on MDR UPEC (40). We believe that this contradicting interaction interpretation is not unusual since it has been also shown that the interaction type of phage-antibiotic is not solely dependent on the MOA of the antibiotic used, but other factors also come into play, such as phage genotype, concentration of both phages and antibiotic used, and other environmental factors (30, 38, 54). Thus, a more holistic approach in phage-antibiotic interaction study that takes crucial factors into account will greatly benefit the design of an effective phage-antibiotic cocktail for translational and therapeutic use in the future.

Regarding the different degrees of bacterial growth revival suppression between each phage-antibiotic pair, we hypothesized that the stability of the antibiotic used in the combination might be responsible for it. This hypothesis was partly supported by phage + PIP and phage + CIP experiments, where bacterial growth revival was significantly suppressed at day 1 but fully emerged to the level comparable to phage alone at day 5. While the instability of PIP, which is a β-lactam antibiotic, is well documented, CIP, a fluoroquinolone, is known to be fairly stable (55, 56). However, it has been shown that the activity of both β-lactam and fluoroquinolone diminished within 10 hours of incubation in the bacterial media, thereby losing the ability to control the growth of the bacteria especially at the level below MIC (57). This could be an explanation why, in our study, the activity of both antibiotics was pronounced only at the early time point and hence could not effectively suppress the bacterial growth revival beyond day 1. In contrast, phage + AMK maintained and even exacerbated the growth suppression ability up to day 5. This could be explained by the fact that AMK, an aminoglycoside antibiotic, is highly stable in various conditions, including in the bacterial growth media (57), resulting in a longer effect that facilitates the PAS effect. Information regarding antibiotic stability will play a crucial role in designing the phage-antibiotic regime in the future, especially in the aspect of antibiotic redosing to maintain the effective dose of those that favor PAS outcomes. Thus, a more comprehensive temporal study of phage-antibiotic combinations would provide a clearer picture of how each antibiotic influences the ability of phage-antibiotic combinations to prevent the bacterial growth revival for long-term use.

Another limitation worth mentioning is that this study focused on the effect of phage-antibiotics in suppressing the growth revival of the UPEC regardless of the information about genetic analyses of emerging bacteria. A previous study shed light on the effect of phage-antibiotic combinations in preventing not only phage resistance emergence but also antibiotic resistance emergence (58). The study conducted in *Staphylococcus aureus* KACC 13236 found that the resistant mutant emerged when using either phage SA11 or ciprofloxacin alone. However, neither antibiotic nor phage resistance was observed in the phage-antibiotic combination group. Similarly, *in vitro* and *in vivo* studies of phage-antibiotics in clinical isolates of *Klebsiella pneumoniae* also revealed that the combination could suppress the rate of either phage- or antibiotic-resistant emergence (39). These imply that the combination treatment is effective in preventing both the emergence of antibiotic and phage resistance. Whether or not the stoichiometric niches of PAS observed in this study could provide better outcomes in preventing the emergence of phage and antibiotic resistance in UPEC should also be considered important experiments in the future for promoting the sustainable use of both phages and antibiotics for therapeutic purposes.

## SUPPLEMENTAL MATERIAL

Supplemental Figure 1 spectrum.00889-23-s0001.tif Lysogeny activation on 10 Killian-resistant isolates

Supplemental figure legends spectrum.00889-23-s0002.docx Supplemental figure legends

Supplemental Figure 2 spectrum.00889-23-s0003.tif Intergenomic similarity values of phage Killian and the closely-related phages as selected from BLAST search

Supplemental Figure 3 spectrum.00889-23-s0004.tif Interaction plot analysis of Killian-PIP combination

Table S1 spectrum.00889-23-s0005.pdf Functional annotation based on protein structures through protein data bank (PDB) and structure predictions through AlphaFold.

ASM does not own the copyrights to Supplemental Material that may be linked to, or accessed through, an article. The authors have granted ASM a non-exclusive, world-wide license to publish the Supplemental Material files. Please contact the corresponding author directly for reuse.
