# Supplementary figures and images for: A novel coli myophage and antibiotics synergistically inhibit the growth of the uropathogenic E. coli strain CFT073 in stoichiometric niches
Source: Microbiol Spectr. 2023 Sep 21;11(5):e00889-23. doi: 10.1128/spectrum.00889-23 (PMC10580823; doi:10.1128/spectrum.00889-23)

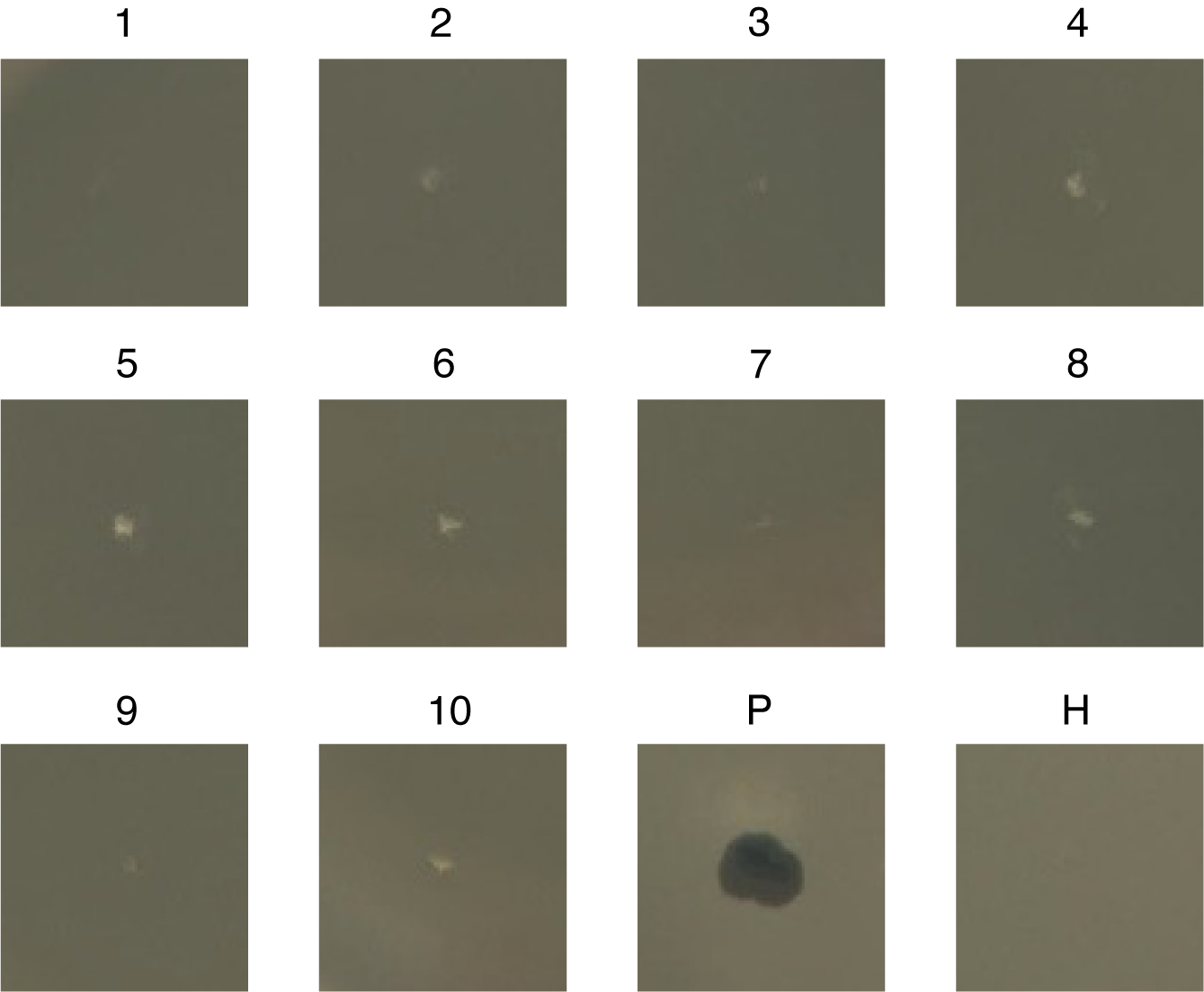

Supplement: Supplemental Figure 1 — Lysogeny activation on 10 Killian-resistant isolates [file spectrum.00889-23-s0001.tif]

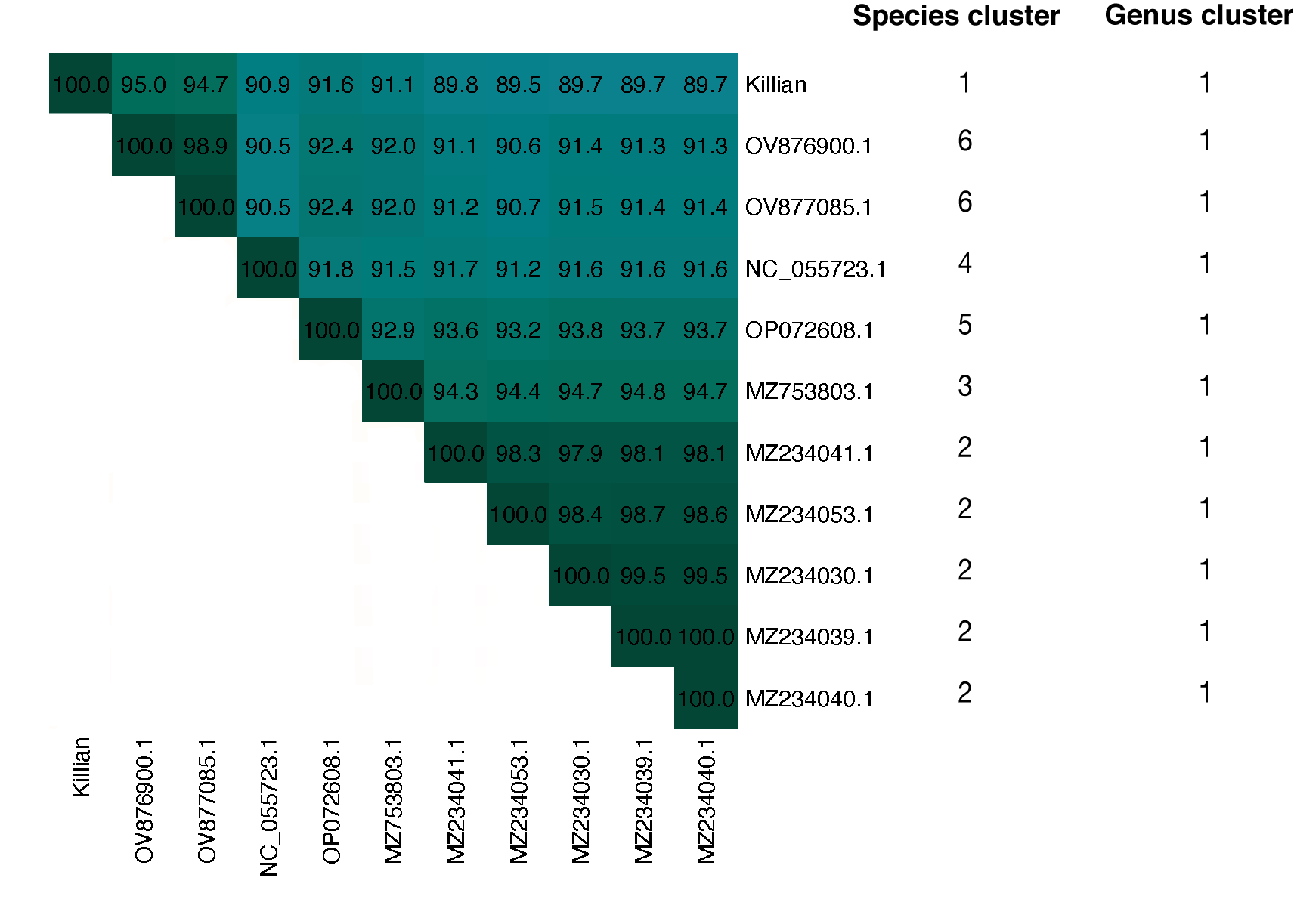

Supplement: Supplemental Figure 2 — Intergenomic similarity values of phage Killian and the closely-related phages as selected from BLAST search [file spectrum.00889-23-s0003.tif]

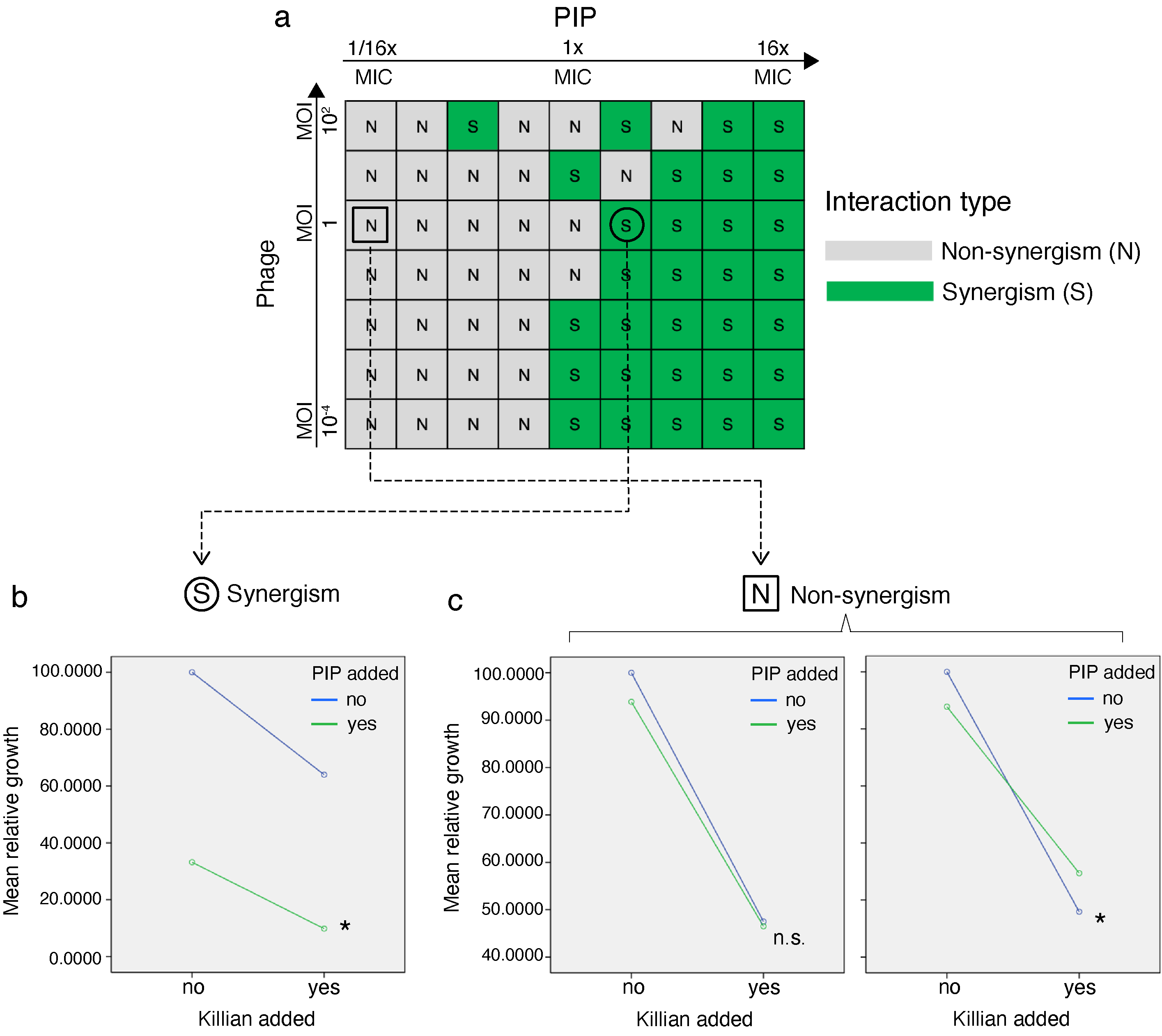

Supplement: Supplemental Figure 3 — Interaction plot analysis of Killian-PIP combination [file spectrum.00889-23-s0004.tif]
