# Supplementary material for: A novel coli myophage and antibiotics synergistically inhibit the growth of the uropathogenic E. coli strain CFT073 in stoichiometric niches
Source: Microbiol Spectr. 2023 Sep 21;11(5):e00889-23. doi: 10.1128/spectrum.00889-23 (PMC10580823; doi:10.1128/spectrum.00889-23)
Supplement: Supplemental figure legends [file spectrum.00889-23-s0002.docx]

**Supplemental figure legends**

**Figure S1** **Lysogeny activation on 10 Killian-resistant isolates (1 to 10).** The isolates were exposed to UV light for 10 seconds and then stabbed on bacterial lawns of UPEC strain CFT073. The visible plaques are considered as the presence of prophage in the phage-resistant strains. The original host strain alone (UPEC strain CFT073) was designated H and the high titer of phage lysates as a positive control was designated P.

**Figure S2 Intergenomic similarity values of phage Killian and the closely-related phages as selected from BLAST search.** The color-coding heatmap as created by VIRIDIC on the left provides a quick view of the clustering of the phage genomes, based on intergenomic similarity: the darker the color, the higher the similarities between the genomes. The numbers represent the first-decimal-rounded similarity values for each genome pair. Based on the similarity values, phages are classified into genus and species, as shown in the table on the right.

**Figure S3 Interaction plot analysis of Killian-PIP combination.** (a) The summary of interaction types of Killian in combination with PIP against UPEC strain CFT073 at various conditions. Interaction plots of Killian-PIP that define (b) synergism, (c) non-synergism, based on relative growth at 24-hour time point. Two-way ANOVA was used for statistical significance testing. *P<0.05; n.s., not significant.
