# Supplementary material for: A novel coli myophage and antibiotics synergistically inhibit the growth of the uropathogenic E. coli strain CFT073 in stoichiometric niches
Source: Microbiol Spectr. 2023 Sep 21;11(5):e00889-23. doi: 10.1128/spectrum.00889-23 (PMC10580823; doi:10.1128/spectrum.00889-23)
Supplement: Table S1 — Functional annotation based on protein structures through protein data bank (PDB) and structure predictions through AlphaFold. [file spectrum.00889-23-s0005.pdf]

Supplemental Table S1 Functional annotation based on protein structures through protein data bank (PDB) and structure predictions through AlphaFold.

ORF	Predicted function	Sequence similarlity	Accession no.	Database	E-value	Predited function through protein structure	Accession no.	Database	E-value
ORF1	Tail fiber protein	Tail fiber protein [*Escherichia* phage vB_EcoM_Shinka]	QXV73019	NCBI	0.00E+00	Major tail fiber protein *Klebsiella pneumoniae*	A0A0D3RK47-F1	AlphaFold	1.20E-289
ORF2	Tail fiber protein	Tail fiber protein [*Escherichia* phage UGKSEcP2]	CAH1615638	NCBI	0.00E+00	Phage tail protein	A0A5N5XX32-F1	AlphaFold	3.50E-11
ORF3	Holin protein	Holin protein [*Escherichia* phage vB_EcoM_SP1]	QLF80826	NCBI	0.00E+00	Holin; phage, lysis inhibition, VIRAL PROTEIN; 2.3A {*Enterobacteria* phage T4}	6PXE_T	PDB	2.10E-86
ORF4	DNA-binding transcriptional regulator	PHAGE_Escher_CF2_NC_041919: DNA-binding transcriptional regulator; phage(gi100278)	PP_00004	PHASTER	1.03E-58	10 KDA Anti-Sigma Factor; All-alpha, Helix-Turn-Helix, Coiled-Coil, TRANSCRIPTION; NMR {*Enterobacteria* phage T4}	1JR5_B	PDB	3.10E-48
ORF5	Hypothetical protein	Hypothetical protein pSs1_00259 [Shigella phage pSs-1]	YP_009111067	NCBI	2.50E-26	Uncharacterized protein	A0A3R7FSJ2-F1	AlphaFold	2.20E-40
ORF6	Anti-restriction nuclease	Anti-restriction nuclease [*Shigella* phage ESh25]	URY13403	NCBI	0.00E+00	Anti-restriction endonuclease; DNA MIMIC, GENE REGULATION, VIRAL PROTEIN; 1.9A {*Enterobacteria* phage T4}	3WX4_A	PDB	2.20E-61
ORF7	Anti-restriction nuclease	Anti-restriction nuclease [*Escherichia* phage YUEEL01]	YP_010074974	NCBI	0.00E+00	Uncharacterized protein	A0A2M9X366-F1	AlphaFold	4.90E-37
ORF8	Anti-restriction nuclease	Anti-restriction nuclease [*Escherichia* phage UPEC01]	QQG30937.1	NCBI	2.00E-95	Uncharacterized protein	A0A2M9X350-F1	AlphaFold	1.80E-50
ORF9	Hypothetical protein	MULTISPECIES hypothetical protein [Bacteria YP_002854591.1]	WP_015983769	NCBI	2.60E-27	Uncharacterized protein	A0A1B8Y4R0-F1	AlphaFold	2.30E-19
ORF10	Anti-restriction nuclease	Anti-restriction nuclease [*Citrobacter* phage vB_CroM_CrRp10]	WP_088209660	NCBI	0.00E+00	Uncharacterized protein	A0A2M9X3D9-F1	AlphaFold	4.30E-46
ORF11	lysozyme	PHAGE_Shigel_Sf24_NC_042078: lysozyme; phage(gi100110)	PP_00013	PHASTER	1.36E-150	Middle transcription regulatory protein motA	6K4Y_M	PDB	6.70E-88
ORF12	Hypothetical protein	Hypothetical protein D862_gp013 [*Escherichia* phage vB_EcoM_ACG-C40]	WP_016054252	NCBI	1.40E-45	Uncharacterized protein	A0A2M9X343-F1	AlphaFold	7.90E-43
ORF13	Flap endonuclease	PHAGE_Escher_CF2_NC_041919: flap endonuclease; phage(gi100267)	PP_00015	PHASTER	2.57E-24	Flagellar motor protein MotA	A0A2M9X3B5-F1	AlphaFold	5.90E-30
ORF14	DNA topoisomerase	DNA topoisomerase *Shigella* phage ESh26	URY13538	NCBI	0.00E+00	DNA topoisomerase	Q8X1U1-F1	PDB	1.40E-163

ORF	Predicted function	Sequence similarlity	Accession no.	Database	E-value	Predited function through protein structure	Accession no.	Database	E-value
ORF15	Acridine resistance protein	Acridine resistance protein [*Tequatrovirus* RB14]	WP_015996044	NCBI	2.30E-27	Sugar ABC transporter permease *Bacillus cereus*	A0A2M9X354-F1	AlphaFold	5.40E-31
ORF16	Nuclear disruption protein	Nuclear disruption protein [*Escherichia* phage ime09]	WP_016059142	NCBI	0.00E+00	Naphthalene 1,2-dioxygenase	A0A0G9GXV6-F1	AlphaFold	8.00E-60
ORF17	Naphthalene 1 2-dioxygenase	Naphthalene 1 2-dioxygenase [*Escherichia* phage]	YP_009210184	NCBI	6.90E-43	Naphthalene 1,2-dioxygenase	A0A2M9X363-F1	AlphaFold	1.20E-29
ORF18	Putative outer membrane protein	Putative outer membrane protein [*Yersinia* phage PST]	YP_009153867	NCBI	1.90E-16	Naphthalene 1,2-dioxygenase	A0A2M9X355-F1	AlphaFold	2.70E-16
ORF19	Predicted periplasmic protein	Predicted periplasmic protein [*Escherichia* phage ime09]	YP_007004641	NCBI	1.50E-38	Uncharacterized protein	A0A2M9X342-F1	AlphaFold	4.10E-39
ORF20	Hypothetical protein	Hypothetical protein [*Shigella* phage CT01]	UDY80661	NCBI	5.70E-15	Naphthalene 1,2-dioxygenase	A0A2M9X3C8-F1	AlphaFold	4.30E-18
ORF21	Hypothetical protein	Hypothetical protein F412_gp006 [*Escherichia* phage wV7]	YP_007005010	NCBI	7.60E-13	Naphthalene 1,2-dioxygenase	A0A2M9X357-F1	AlphaFold	3.60E-22
ORF22	Hypothetical protein	Hypothetical protein SA20RB_266 [*Escherichia* phage vB_EcoM_SA20RB]	UIU28212	NCBI	2.80E-34	Uncharacterized protein	A0A2M9X3I1-F1	AlphaFold	2.70E-30
ORF23	Hypothetical protein	RB32ORF266c hypothetical protein [*Tequatrovirus* RB14]	YP_002854606	NCBI	8.60E-28	Uncharacterized protein	A0A6P6QUB7-F1	AlphaFold	0.000042
ORF24	Endonuclease	Endonuclease [*Escherichia* phage EcNP1]	YP_010068172	NCBI	0.00E+00	Uncharacterized protein	A0A2M9X332-F1	AlphaFold	6.90E-72
ORF25	Hypothetical protein	Hypothetical protein [*Escherichia* phage vB_EcoM_Nami]	QXV73418	NCBI	0.00E+00	Uncharacterized protein	A0A401NZN3-F1	AlphaFold	1.20E-20
ORF26	ATP-dependent helicase	PHAGE_Shigel_Sf24_NC_042078: ATP-dependent helicase; phage(gi100124)	PP_00027	PHASTER	4.46E-39	Uncharacterized protein	A0A077NTD4-F1	AlphaFold	1.40E-24
ORF27	Transcription regulators	Helix-turn-helix domain-containing protein [*Enterobacteria* phage vB_EcoM_IME339]	YP_010094422	NCBI	0.00E+00	RIIB Protector from prophage-induced early lysis	A0A120DEX4-F1	AlphaFold	3.80E-88
ORF28	Histidine kinase-like ATPase	[HATPase super family] cl00075 (PSSM 412147) Histidine kinase-like ATPase domain	YP_010068176	NCBI conserve domain	0.00E+00	TNF receptor-associated protein 1; Chaperone, ATPase, ATP binding,	4IPE_B	PDB	8.90E-16

ORF	Predicted function	Sequence similarlity	Accession no.	Database	E-value	Predited function through protein structure	Accession no.	Database	E-value
ORF29	Recombination endonuclease subunit	PHAGE_Shigel_Sf24_NC_042078: recombination endonuclease subunit D12; phage(gi100127)	PP_00030	PHASTER	8.73E-42	Uncharacterized protein	A0A2M9X329-F1	AlphaFold	2.20E-35
ORF30	ATP-binding protein	DNA topoisomerase subunit DNA-dependent ATPase [Tequatrovirus RB14]	WP_015983554	NCBI	0.00E+00	DNA topoisomerase 2-alpha	6ZY7_B	PDB	2.90E-97
ORF31	Hypothetical protein	Hypothetical protein JK23_00004 [Shigella phage JK23]	QEG05043	NCBI	0.00E+00	Uncharacterized protein	A0A8B5SB19-F1	AlphaFold	1.80E-30
ORF32	ssDNA binding protein	PHAGE_Escher_slur04_NC_042130: ssDNA binding protein; phage(gi100259)	PP_00033	PHASTER	2.24E-81	Uncharacterized protein	A0A2M9X331-F1	AlphaFold	6.00E-58
ORF33	Putative zinc ribbon domain-containing protein	Putative zinc ribbon domain-containing protein [*Escherichia* phage vB_EcoM_Nami]	QXV73513	NCBI	1.30E-24	Zinc ribbon domain-containing protein	A0A523D4P7-F1	AlphaFold	4.50E-16
ORF34	Hypothetical protein	Hypothetical protein UGKSECP2_00021 [*Escherichia* phage UGKSEcP2]	CAH1615308	NCBI	0.00E+00	Uncharacterized protein	A0A2M9X347-F1	AlphaFold	8.90E-56
ORF35	Modifier of suppressor tRNAs	Modifier of suppressor tRNAs [*Escherichia* phage vB_EcoM-CHD2BS1]	QZI80302	NCBI	1.30E-44	Cytochrome C biogenesis protein CcmE	A0A2M9X323-F1	AlphaFold	2.40E-33
ORF36	MotB-like transcriptional regulator	MotB-like transcriptional regulator [*Escherichia* phage UGKSEcP2]	CAH1615310	NCBI	0.00E+00	Uncharacterized protein	A0A2M9X396-F1	AlphaFold	5.80E-69
ORF37	Modifier of transcription	[motB Superfamily] cl38873 (PSSM 365749) transcription regulation-related protein	CAH1615311	NCBI conserve domain	0.00E+00	Uncharacterized protein	A0A2M9X320-F1	AlphaFold	7.10E-98
ORF38	Cell wall hydrolase	PHAGE_Escher_HY03_NC_031047: cell wall hydrolase; phage(gi100184)	PP_00039	PHASTER	1.33E-116	Uncharacterized protein	A0A2M9X333-F1	AlphaFold	3.80E-80
ORF39	3'-5' exoribonuclease	3'-5' exoribonuclease [*Escherichia* phage vB_EcoM_OE5505]	YP_010072298	NCBI	0.00E+00	3'-5' exonuclease ERI1	1ZBU_B	PDB	1.70E-15
ORF40	Hypothetical protein	Hypothetical protein	EEX9354875	NCBI	0.00E+00	Dextranase	A0A2M9X336-F1	AlphaFold	0.0000019
ORF41	Dextranase	Dextranase [Yersinia phage phiD1]	YP_009149254	NCBI	0.00E+00	Dextranase	A0A2M9X336-F1	AlphaFold	7.90E-51
ORF42	Hypothetical protein	gp30 [*Shigella* phage pSs-1]	YP_009110837.1	NCBI	0.00E+00	gp30 [*Shigella* phage pSs-1]	YP_009110837.1	BlastX	1.00E-53

ORF	Predicted function	Sequence similarlity	Accession no.	Database	E-value	Predited function through protein structure	Accession no.	Database	E-value
ORF43	AAA family ATPase	AAA family ATPase [*Escherichia* phage vB_EcoM_G50]	YP_010069850.1	NCBI	0.00E+00	ATP-dependent DNA helicase	3UPU_B	PDB	2.70E-46
ORF44	Hypothetical protein	Hypothetical protein UGKSECP2_00031 [*Escherichia* phage UGKSEcP2]	CAH1615318	NCBI	0.00E+00	Uncharacterized protein	A0A2M9X3A4-F1	AlphaFold	1.20E-26
ORF45	Hypothetical protein	Anti-sigma factor [*Escherichia* phage vB_EcoM_SA21RB]	EEX9354880	NCBI	0.00E+00	Uncharacterized protein	A0A2M9X337-F1	AlphaFold	2.00E-82
ORF46	Putative adenylribosylating enzyme	Putative adenylribosylating enzyme [*Shigella* phage Shfl2]	YP_004414919	NCBI	0.00E+00	Type III effector HopU1; ADP-ribosyltransferase	3U0J_B	PDB	0.00003
ORF47	RNA polymerase ADP-ribosylase	RNA polymerase ADP-ribosylase [*Escherichia* phage UGKSEcP2]	CAH1615321	NCBI	0.00E+00	ADP-RIBOSYLTRANSFERASE	1QS1_B	PDB	1.40E-07
ORF48	Central straight tail fiber	PHAGE_Escher_slur04_NC_042130: central straight tail fiber; phage(gi100275)	PP_00049;	PHASTER	4.51E-38	Uncharacterized protein	A0A2M9X311-F1	AlphaFold	2.50E-24
ORF49	TraG-like protein	PHAGE_Shigel_SHFML_26_NC_031011: TraG-like protein; phage(gi100120)	PP_00050	PHASTER	3.20E-111	Uncharacterized protein	A0A2M9X321-F1	AlphaFold	1.00E-99
ORF50	Molybdenum ABC transporter	Molybdenum ABC transporter [*Enterobacteria* phage phiC600P9]	QNI20112.1	NCBI	6.20E-30	hypothetical protein	WP_176393784.1	BlastX	3.00E-29
ORF51	Putative Srh transcription modulator	Putative Srh transcription modulator [*Shigella* phage Shfl2]	YP_004414924	NCBI	8.10E-39	S-ribosylhomocysteinase	A0A2M9X308-F1	AlphaFold	3.60E-35
ORF52	Transcription modulator	Transcription modulator under heat shock [*Shigella* phage Sfk20]	QPP47073	NCBI	0.00E+00	Uncharacterized protein	A0A2M9X328-F1	AlphaFold	2.90E-97
ORF53	Hypothetical protein	Hypothetical protein [*Shigella* phage ESh26]	URY13580	NCBI	3.20E-30	Uncharacterized protein	A0A2M9X326-F1	AlphaFold	2.00E-25
ORF54	Hypothetical protein	mrh.2 hypothetical protein [*Tequatrovirus* RB14]	WP_015995894	NCBI	4.40E-43	Uncharacterized protein	A0A2M9X312-F1	AlphaFold	5.40E-34
ORF55	Small outer capsid protein	Small outer capsid protein [*Escherichia* phage W143]	QWV60377	NCBI	0.00E+00	Small outer capsid protein; Bacteriophage T4	5VF3_X	PDB	4.50E-51
ORF56	dCTP pyrophosphatase	dCTP pyrophosphatase [*Escherichia* phage vB_EcoM_WL-3]	QOQ37406	NCBI	0.00E+00	dCTP pyrophosphatase	A0A2M9X303-F1	AlphaFold	2.80E-40

ORF	Predicted function	Sequence similarlity	Accession no.	Database	E-value	Predited function through protein structure	Accession no.	Database	E-value
ORF57	HNH endonuclease	HNH endonuclease [*Escherichia* phage vB_EcoM_IME537]	YP_010071070	NCBI	0.00E+00	HNH endonuclease family protein	A0A0U1P513-F1	AlphaFold	1.30E-49
ORF58	Hypothetical protein	Hypothetical protein [*Escherichia* phage vB_EcoM_Nami]	QXV73496	NCBI	7.10E-37	Uncharacterized protein	A0A2M9X309-F1	AlphaFold	1.40E-32
ORF59	DNA primase	DNA primase [*Escherichia* phage EC121]	YP_010067719	NCBI	0.00E+00	DNA primase	A0A2E5AWE1-F1	AlphaFold	7.10E-128
ORF60	Major capsid domain-containing protein	Major capsid domain-containing protein [*Escherichia* phage teqsoen]	QHR63884	NCBI	7.90E-21	Phage capsid protein	A0A2M9X3C2-F1	AlphaFold	1.40E-09
ORF61	Putative major capsid protein	Putative major capsid protein [*Salmonella* phage pSe_SNUABM_01]	YP_010075302	NCBI	0.00E+00	putative major capsid protein	YP_010075302.1	BLastX	2.00E-150
ORF62	Spackle periplasmic protein	Spackle periplasmic protein [*Shigella* phage SHFML-26]	YP_009279032.1	NCBI	0.00E+00	Protein spackle; APOBEC, deaminase, HYDROLASE	6X6O_A	PDB	1.70E-47
ORF63	Hypothetical protein	Hypothetical protein UGKSECP1_00160 [*Escherichia* phage UGKSEcP1]	CAH1615974	NCBI	0.00E+00	Uncharacterized protein	A0A2M9X302-F1	AlphaFold	3.90E-37
ORF64	Discriminator of mRNA degradation	Discriminator of mRNA degradation [*Escherichia* phage EC121]	YP_010067724	NCBI	1.40E-35	Dmd discriminator of mRNA degradation	5I8J_A	PDB	3.20E-35
ORF65	AAA family ATPase	AAA family ATPase [*Shigella* phage pSs-1]	YP_009110859	NCBI	0.00E+00	DNA primase	A0A2E5AWC2-F1	AlphaFold	3.60E-177
ORF66	Head vertex assembly chaperone	Head vertex assembly chaperone [*Shigella* phage CM8]	YP_010075847.	NCBI	0.00E+00	Uncharacterized protein	A0A2M9X2Z2-F1	AlphaFold	6.90E-42
ORF67	Putative UvsX recA-like recombination protein	Putative UvsX recA-like recombination protein [*Shigella* phage Shfl2]	YP_004414943.1	NCBI	0.00E+00	Recombinase RecA	A0A3M1U0S8-F1	AlphaFold	8.20E-119
ORF68	Putative beta-glucosyl-HMC-alpha-glucosyl-transferase	Putative beta-glucosyl-HMC-alpha-glucosyl-transferase [*Escherichia* phage HY01]	YP_009148490	NCBI	0.00E+00	Glycosyl transferase	5TZ8_B	PDB	9.60E-19
ORF69	Thymidylate synthase	Thymidylate synthase [*Escherichia* phage UGKSEcP2]	CAH1615343	NCBI	0.00E+00	THYMIDYLATE SYNTHASE	1TIS_A	PDB	1.90E-44
ORF70	Immunity to superinfection membrane protein	Immunity to superinfection membrane protein [*Escherichia* phage vB_EcoM_SYGD1]	QUD16027	NCBI	0.00E+00	Superinfection immunity protein	A0A1H2RDW5-F1	AlphaFold	2.70E-21

ORF	Predicted function	Sequence similarlity	Accession no.	Database	E-value	Predited function through protein structure	Accession no.	Database	E-value
ORF71	Immunity protein	Immunity protein [*Escherichia* phage vB_EcoM_CE1]	URG13343	NCBI	0.00E+00	Iduronate sulfatase	A0A7Z1HN92-F1	AlphaFold	1.70E-70
ORF72	Major tail protein	PHAGE_Escher_HY03_NC_031047: major tail protein; phage(gi100145)	PP_00073	PHASTER	1.60E-45	Uncharacterized protein	A0A1F8MHP9-F1	AlphaFold	2.40E-10
ORF73	DNA polymerase	DNA polymerase [*Escherichia* phage UGKSEcP2]	CAH1615347.1	NCBI	0.00E+00	DNA polymerase	3QEX_A	PDB	8.40E-109
ORF74	Translational repressor RegA	Translational repressor RegA [*Escherichia* phage T4]	YP_002854006	NCBI	0.00E+00	Translational repressor RegA	A0A843GNY1-F1	AlphaFold	1.00E-29
ORF75	DNA polymerase clamp loader subunit A	DNA polymerase clamp loader subunit A [*Escherichia* phage vB_EcoM-CHD94UKE2]	QZI80988	NCBI	0.00E+00	DNA polymerase	A0A2M9X306-F1	AlphaFold	2.70E-78
ORF76	Putative replication factor	Putative replication factor C small subunit [*Escherichia* phage JLBYU24]	UGO55400	NCBI	0.00E+00	Replication factor C subunit 3	A0A2U1PWJ8-F1	AlphaFold	9.40E-139
ORF77	Phage-associated sliding clamp	Phage-associated sliding clamp DNA polymerase accessory protein [*Escherichia* phage ime09]	YP_007004436.1	NCBI	0.00E+00	SLIDING CLAMP	1B77_B	PDB	4.50E-65
ORF78	RNA polymerase binding protein	RNA polymerase binding protein [*Escherichia* phage vB_EcoM_SA20RB]	UIU27997	NCBI	0.00E+00	RNA polymerase-binding protein	A0A2M9X2Z1-F1	AlphaFold	3.40E-41
ORF79	Prohead protease	PHAGE_Shigel_SHFML_26_NC_031011: prohead protease; phage(gi100150)	PP_00080	PHASTER	6.17E-40	Uncharacterized protein	A0A2M9X2X9-F1	AlphaFold	8.10E-31
ORF80	AAA family ATPase	AAA family ATPase [*Escherichia* phage ime09]	YP_007004439	NCBI	0.00E+00	AAA_23 domain-containing protein	A0A2D6WZB4-F1	AlphaFold	8.20E-194
ORF81	Tail length tape-measure protein	PHAGE_Escher_UFV_AREG1_NC_031030: tail length tape-measure protein; phage(gi100057)	PP_00082	PHASTER	9.03E-43	Uncharacterized protein	A0A2M9X2Y3-F1	AlphaFold	8.20E-34
ORF82	Endolysin	PHAGE_Escher_slur04_NC_042130: endolysin; phage(gi100033)	PP_00083	PHASTER	3.05E-58	Uncharacterized protein	A0A2M9X365-F1	AlphaFold	1.00E-40
ORF83	Recombination-related endonuclease	Recombination-related endonuclease [*Enterobacteria* phage phiC600P9]	QNI20146	NCBI	0.00E+00	Exonuclease	3THO_B	PDB	1.10E-28
ORF84	Homing endonuclease	Homing endonuclease [*Klebsiella* phage KP185]	UNY41130	NCBI	0.00E+00	Intron-associated endonuclease	1MK0_A	PDB	8.40E-08

ORF	Predicted function	Sequence similarlity	Accession no.	Database	E-value	Predited function through protein structure	Accession no.	Database	E-value
ORF85	Alpha glucosyltransferase	Alpha glucosyltransferase [*Shigella* phage Sf24]	YP_009619268	NCBI	0.00E+00	DNA alpha-glucosyltransferase	1XV5_A	PDB	1.10E-40
ORF86	Metallopeptidase	PHAGE_Escher_CF2_NC_041919: metallopeptidase; phage(gi100188)	PP_00088	PHASTER	3.96E-44	Uncharacterized protein	A0A2M9X349-F1	AlphaFold	1.10E-28
ORF87	Hypothetical protein	MULTISPECIES a-gt.4 family protein	WP_015969224	NCBI	0.00E+00	Uncharacterized protein	A0A2M9X2X2-F1	AlphaFold	1.00E-30
ORF88	Hypothetical protein	a-gt.5 hypothetical protein [*Escherichia* phage T4]	NP_049678.1	NCBI	2.00E-43	Uncharacterized protein	A0A2M9X2Y0-F1	AlphaFold	5.00E-30
ORF89	RNA polymerase sigma factor	RNA polymerase sigma factor [*Escherichia* phage vB_EcoM_112]	YP_009030673	NCBI	0.00E+00	Sigma factor for late transcription	A0A2D5MTK9-F1	AlphaFold	4.50E-62
ORF90	Cell wall hydrolase	PHAGE_Escher_CF2_NC_041919: cell wall hydrolase; phage(gi100184)	PP_00092	PHASTER	9.95E-61	Uncharacterized protein	A0A2M9X2Y7-F1	AlphaFold	8.20E-34
ORF91	Recombination endonuclease VII	Recombination endonuclease VII [*Escherichia* phage HP3]	YP_010228891	NCBI	4.20E-40	Uncharacterized protein	A0A2M9X2Y5-F1	AlphaFold	1.50E-31
ORF92	Hypothetical protein	Hypothetical protein Shfl2p071 [*Shigella* phage Shfl2]	YP_004414968	NCBI	0.00E+00	Uncharacterized protein	A0A2M9X2X3-F1	AlphaFold	7.10E-48
ORF93	Hypothetical protein	Hypothetical protein Shfl2p072 [*Shigella* phage Shfl2]	YP_004414969	NCBI	2.10E-41	Uncharacterized protein	A0A2M9X356-F1	AlphaFold	2.30E-40
ORF94	Hypothetical protein	Hypothetical protein KMC13_gp181 [*Escherichia* phage vB_EcoM_IME537]	YP_010071109	NCBI	3.50E-23	Uncharacterized protein	A0A3L1NQP6-F1	AlphaFold	9.60E-21
ORF95	Hypothetical protein	Hypothetical protein PHAGINATOR_72 [*Shigella* phage vB_SboM_Phaginator]	UGO46745	NCBI	0.00E+00	Uncharacterized protein	A0A2M9X2Y6-F1	AlphaFold	1.40E-40
ORF96	Chaperone	Chaperone for long tail fiber formation [*Enterobacteria* phage T6]	YP_010067224	NCBI	1.10E-31	Uncharacterized protein	A0A2M9X2W1-F1	AlphaFold	1.70E-28
ORF97	NrdH glutaredoxin	NrdH glutaredoxin [*Escherichia* phage T4]	NP_049686.1	NCBI	0.00E+00	Glutaredoxin	A0A2E4W6R2-F1	AlphaFold	1.10E-12
ORF98	Hypothetical protein	Hypothetical protein BI058_gp077 [*Shigella* phage SHBML-50-1]	YP_009288443	NCBI	1.90E-29	Uncharacterized protein	A0A2M9X2W2-F1	AlphaFold	4.90E-36

ORF	Predicted function	Sequence similarlity	Accession no.	Database	E-value	Predited function through protein structure	Accession no.	Database	E-value
ORF99	Anaerobic ribonucleoside-triphosphate reductase activating protein	Anaerobic ribonucleoside-triphosphate reductase activating protein [*Escherichia* phage ime09]	YP_007004459.1	NCBI	0.00E+00	Anaerobic ribonucleoside-triphosphate reductase-activating protein	A0A1I1MUF4-F1	AlphaFold	3.70E-55
ORF100	Endolysin	PHAGE_Escher_ST0_NC_041990: endolysin; phage(gi100033)	PP_00103	PHASTER	0.00E+00	ANAEROBIC RIBONUCLEOTIDE-TRIPHOSPHATE REDUCTASE LARGE CHAI	1H7B_A	PDB	1.00E-85
ORF101	Putative homing endonuclease	Putative homing endonuclease [*Escherichia* phage TadeuszReichstein]	QXV84933	NCBI	0.00E+00	Uncharacterized protein	A0A7Z1KKI9-F1	AlphaFold	6.20E-53
ORF102	Endonuclease domain-containing protein	Endonuclease domain-containing protein [*Escherichia coli*]	WP_015983616	NCBI	0.00E+00	Endonuclease VII	A0A2M9X2X8-F1	AlphaFold	2.30E-38
ORF103	Peptidase	Peptidase [*Yersinia* phage phiD1]	YP_009149322	NCBI	0.00E+00	Peptidase	A0A2M9X2X6-F1	AlphaFold	2.40E-44
ORF104	Ribonucleotide reductase	Ribonucleotide reductase [*Escherichia* phage UGKSEcP2]	CAH1615376.1	NCBI	0.00E+00	Uncharacterized protein	A0A2P1V6C8-F1	AlphaFold	4.60E-20
ORF105	Hypothetical protein	Hypothetical protein UGKSECP2_00090 [*Escherichia* phage UGKSEcP2]	CAH1615377	NCBI	0.00E+00	Uncharacterized protein	A0A2M9X2W4-F1	AlphaFold	9.20E-40
ORF106	Phage-associated thioredoxin	Phage-associated thioredoxin [*Bacillus cereus*]	WP_016059029	NCBI	0.00E+00	thioredoxin reductase	A0A1S3DS09-F1	AlphaFold	1.30E-75
ORF107	Thioredoxin	Thioredoxin [Shigella phage ESh28]	URY14166	NCBI	0.00E+00	Uncharacterized protein	A0A2M9X2X5-F1	AlphaFold	7.70E-31
ORF108	Thioredoxin	Thioredoxin [*Enterobacteria* phage phiC600P9]	QNI20173.1	NCBI	0.00E+00	Uncharacterized protein	A0A2M9X2V2-F1	AlphaFold	9.70E-44
ORF109	Thioredoxin	Thioredoxin [*Escherichia* phage JEP6]	QOC55280	NCBI	0.00E+00	Uncharacterized protein	A0A2M9X334-F1	AlphaFold	3.20E-190
ORF110	Thioredoxin	Thioredoxin [*Escherichia* phage teqhad]	YP_010074292	NCBI	0.00E+00	Uncharacterized protein	A0A2M9X3G2-F1	AlphaFold	2.50E-154
ORF111	Thioredoxin	Thioredoxin [*Escherichia* phage vB_EcoM_Shinka]	QXV73054	NCBI	0.00E+00	Uncharacterized protein	A0A2M9X2V4-F1	AlphaFold	9.10E-228
ORF112	Thioredoxin	Thioredoxin [*Enterobacteria* phage vB_EcoM_IME340]	YP_010066561	NCBI	0.00E+00	Uncharacterized protein	A0A2M9X2V5-F1	AlphaFold	3.30E-13

ORF	Predicted function	Sequence similarlity	Accession no.	Database	E-value	Predited function through protein structure	Accession no.	Database	E-value
ORF113	Putative Mur ligase domain-containing protein	Putative Mur ligase domain-containing protein [*Escherichia* phage vB_EcoM_IME537]	YP_010071132	NCBI	0.00E+00	Uncharacterized protein	A0A0G9GWP0-F1	AlphaFold	1.40E-37
ORF114	Thioredoxin	Thioredoxin [*Enterobacteria* phage GiZh]	YP_010066301	NCBI	0.00E+00	Uncharacterized protein	A0A0G9GX20-F1	AlphaFold	9.20E-75
ORF115	Thioredoxin	Thioredoxin [*Shigella* phage ESh26]	URY13649	NCBI	0.00E+00	Uncharacterized protein	A0A2M9X2W5-F1	AlphaFold	1.00E-66
ORF116	Thioredoxin	Thioredoxin [*Shigella* phage ESh16]	URY11581	NCBI	0.00E+00	AAA domain-containing protein	A0A2M9X335-F1	AlphaFold	1.70E-119
ORF117	Hypothetical protein	DUF4031 domain-containing protein [*Escherichia* phage ECML-134]	YP_009102568	NCBI	0.00E+00	UF4031 domain-containing protein	A0A0F8WIH3-F1	AlphaFold	3.00E-30
ORF118	Hypothetical protein	Hypothetical protein KMC02_gp052 [*Escherichia* phage EcNP1]	YP_010068005.1	NCBI	2.50E-25	Hypothetical protein	YP_010068005.1	BlastX	3.00E-25
ORF119	Thioredoxin	Thioredoxin [*Salmonella* phage GRNsp7]	USW07363	NCBI	0.00E+00	Uncharacterized protein	A0A2M9X2U8-F1	AlphaFold	3.80E-96
ORF120	Signal-peptide domain-containing protein	Signal-peptide domain-containing protein [*Escherichia* phage HY01]	YP_009148542	NCBI	0.00E+00	Uncharacterized protein	A0A2M9X324-F1	AlphaFold	1.00E-47
ORF121	Hypothetical protein	Hypothetical protein KMC02_gp055 [*Escherichia* phage EcNP1]	YP_010068008	NCBI	0.00E+00	Uncharacterized protein	A0A2M9X2U5-F1	AlphaFold	8.90E-114
ORF122	Molybdopterin-guanine dinucleotide biosynthesis protein	Molybdopterin-guanine dinucleotide biosynthesis protein [MobD *Shigella* phage pSs-1]	YP_009110919	NCBI	0.00E+00	Molybdopterin-guanine dinucleotide biosynthesis protein	A0A2M9X3R4-F1	AlphaFold	7.30E-82
ORF123	Putative molybdopterin-guanine dinucleotide biosynthesis	Putative molybdopterin-guanine dinucleotide biosynthesis protein MobD [*Escherichia* phage	UGO55623	NCBI	2.90E-13	Molybdopterin-guanine dinucleotide biosynthesis protein	A0A2M9X2U9-F1	AlphaFold	1.50E-16
ORF124	Hypothetical protein	Hypothetical protein F413_gp150 [*Escherichia* phage ime09]	YP_007004482	NCBI	6.80E-33	Uncharacterized protein	A0A445A0J4-F1	AlphaFold	0.000071
ORF125	Molybdopterin-guanine dinucleotide biosynthesis	Molybdopterin-guanine dinucleotide biosynthesis protein MobD [*Escherichia coli*]	HBN1578523.1	NCBI	1.30E-27	Molybdopterin-guanine dinucleotide	A0A2M9X2U0-F1	AlphaFold	3.50E-33
ORF126	Molybdopterin-guanine dinucleotide biosynthesis	Molybdopterin-guanine dinucleotide biosynthesis protein MobD [*Escherichia coli*]	MBJ0221193	NCBI	2.80E-35	Molybdopterin-guanine dinucleotide biosynthesis protein	A0A2M9X2V7-F1	AlphaFold	5.10E-30

ORF	Predicted function	Sequence similarlity	Accession no.	Database	E-value	Predited function through protein structure	Accession no.	Database	E-value
ORF127	Major tail protein	PHAGE_Escher_CF2_NC_041919: major tail protein; phage(gi100145)	PP_00130	PHASTER	8.94E-39	Molybdopterin-guanine dinucleotide biosynthesis protein	A0A2M9X2V6-F1	AlphaFold	8.90E-38
ORF128	Hypothetical protein	DUF5856 family protein [*Escherichia* phage EcNP1]	YP_010068014	NCBI	0.00E+00	Uncharacterized protein	A0A3N5WBS5-F1	AlphaFold	6.50E-04
ORF129	rI lysis inhibition regulator membrane protein	rI lysis inhibition regulator membrane protein [*Escherichia* phage ime09]	YP_007004487.1	NCBI	0.00E+00	Uncharacterized protein	A0A2M9X325-F1	AlphaFold	1.70E-28
ORF130	Hypothetical protein	rI.1 conserved hypothetical protein [*Escherichia* phage T4]	NP_049718.1	NCBI	1.80E-42	Uncharacterized protein	A0A2M9X2V8-F1	AlphaFold	9.20E-32
ORF131	Thymidine kinase	Thymidine kinase [*Escherichia* phage ECML-134]	YP_009102581	NCBI	0.00E+00	Thymidine kinase	G0UVA0-F1	AlphaFold	3.30E-215
ORF132	Thymidine kinase	Thymidine kinase [*Shigella* phage CM8]	YP_010075911.1	NCBI	0.00E+00	Uncharacterized protein	A0A2M9X314-F1	AlphaFold	1.10E-30
ORF133	Pore-forming tail tip protein	PHAGE_Escher_CF2_NC_041919: pore-forming tail tip protein; phage(gi100139)	PP_00136	PHASTER	2.59E-33	Uncharacterized protein	Q10ZB1-F1	AlphaFold	0.00095
ORF134	Hypothetical protein	Hypothetical protein pSs1_00125 [*Shigella* phage pSs-1]	YP_009110933	NCBI	1.70E-43	Uncharacterized protein	A0A2M9X2T6-F1	AlphaFold	4.60E-26
ORF135	Putative methyltransferase	PHAGE_Shigel_Sf21_NC_042077: putative methyltransferase; phage(gi100107)	PP_00137	PHASTER	1.76E-45	Uncharacterized protein	A0A2M9X2T8-F1	AlphaFold	2.90E-39
ORF136	MazF family toxin-antitoxin system protein;	PHAGE_Shigel_Sf21_NC_042077: MazF family toxin-antitoxin system protein; phage(gi100109)	PP_00139	PHASTER	2.78E-114	RNase III inhibitor	5M3I_B	PDB	1.20E-14
ORF137	Tail fibers protein	PHAGE_Escher_slur03_NC_042129: tail fibers protein; phage(gi100170)	PP_00140	PHASTER	1.53E-79	Uncharacterized protein	A0A2M9X2U6-F1	AlphaFold	3.30E-47
ORF138	Transglycosylase SLT domain-containing protein	Transglycosylase SLT domain-containing protein [*Shigella* phage pSs-1]	YP_009110937.1	NCBI	0.00E+00	SLT domain-containing protein	A0A2M9X2U3-F1	AlphaFold	1.20E-55
ORF139	Site-specific RNase	Site-specific RNase [*Shigella* phage Shfl2]	YP_004415013.1	NCBI	0.00E+00	Ribonuclease	2HX6_A	PDB	4.20E-87
ORF140	Anaerobic ribonucleoside-triphosphate reductase	PHAGE_Shigel_SHFML_26_NC_031011: anaerobic ribonucleoside-triphosphate reductase; phage(gi100213)	PP_00143	PHASTER	1.06E-61	Uncharacterized protein	A0A2M9X315-F1	AlphaFold	1.20E-32

ORF	Predicted function	Sequence similarlity	Accession no.	Database	E-value	Predited function through protein structure	Accession no.	Database	E-value
ORF141	Deoxynucleoside kinase	PHAGE_Escher_HY03_NC_031047: deoxynucleoside kinase; phage(gi100082)	PP_00144	PHASTER	1.21E-57	Uncharacterized protein	A0A2M9X2U4-F1	AlphaFold	8.40E-39
ORF142	Hypothetical protein	Hypothetical protein Vs.5 [*Shigella* phage Shfl2]	YP_004415016	NCBI	1.40E-45	Uncharacterized protein	A0A2M9X2S8-F1	AlphaFold	2.20E-30
ORF143	Autonomous glycyl radical cofactor GrcA	Autonomous glycyl radical cofactor GrcA [*Enterobacteria* phage RB51]	YP_002854079	NCBI	0.00E+00	Autonomous glycyl radical cofactor GrcA	A0A3N4ERI6-F1	AlphaFold	1.00E-46
ORF144	Endoribonuclease	Endoribonuclease [*Escherichia* phage teqdroes]	YP_010074171.1	NCBI	0.00E+00	Uncharacterized protein	A0A2M9X2S3-F1	AlphaFold	4.40E-42
ORF145	Putative endoribonuclease	Putative endoribonuclease [*Escherichia* phage JLBYU31]	UGO55118	NCBI	0.00E+00	Uncharacterized protein	A0A2M9X2S3-F1	AlphaFold	4.40E-42
ORF146	Endonuclease V N-glycosylase UV repair enzyme	Endonuclease V N-glycosylase UV repair enzyme [*Escherichia* phage UGKSEcP2]	CAH1615419	NCBI	0.00E+00	ENDONUCLEASE	2END_A	PDB	1.40E-45
ORF147	Head protein	ipII protein [*Escherichia* phage vB_EcoM_JS09]	YP_009037382.1	NCBI	0.00E+00	Uncharacterized protein	A0A2U9IRP2-F1	AlphaFold	0.00082
ORF148	Endolysin	Endolysin [*Escherichia* phage UGKSEcP2]	CAH1615421	NCBI	0.00E+00	Lysozyme	A0A4Q0Z920-F1	AlphaFold	4.60E-49
ORF149	Nudix hydrolase	Nudix hydrolase [*Shigella* phage ESh17]	URY11946	NCBI	0.00E+00	NUDIX hydrolase	A0A653JWH5-F1	AlphaFold	5.00E-50
ORF150	Membrane protein	Membrane protein [*Escherichia* phage UGKSEcP2]	CAH1615423	NCBI	0.00E+00	Uncharacterized protein	A0A2M9X305-F1	AlphaFold	1.90E-80
ORF151	Sulfurtransferase	Sulfurtransferase [*Escherichia* phage AR1]	YP_009167941	NCBI	0.00E+00	Sulfurtransferase	A0A2M9X2T3-F1	AlphaFold	2.20E-67
ORF152	Sulfurtransferase	Sulfurtransferase [*Shigella* flexneri]	EFW4640016.1	NCBI	0.00E+00	Sulfurtransferase	A0A2M9X2R6-F1	AlphaFold	1.40E-75
ORF153	Putative phosphoserine phosphatase	Putative phosphoserine phosphatase [*Escherichia* phage vB_EcoM_Nami]	QXV73343	NCBI	0.00E+00	Uncharacterized protein	A0A2M9X3I0-F1	AlphaFold	2.10E-118
ORF154	Hypothetical protein	Hypothetical protein [*Escherichia coli*]	WP_074146463	NCBI	0.00E+00	Uncharacterized protein	A0A7Z1KKX4-F1	AlphaFold	3.20E-78

ORF	Predicted function	Sequence similarlity	Accession no.	Database	E-value	Predited function through protein structure	Accession no.	Database	E-value
ORF155	Hypothetical protein	Hypothetical protein UGKSECP2_00141 [*Escherichia* phage UGKSEcP2]	CAH1615428	NCBI	0.00E+00	Hypothetical protein	CAH1615884.1	BlastX	3.00E-112
ORF156	Hypothetical protein	Hypothetical protein KMC02_gp091 [*Escherichia* phage EcNP1]	YP_010068044	NCBI	0.00E+00	Uncharacterized protein	A0A2M9X2R3-F1	AlphaFold	8.60E-50
ORF157	Hypothetical protein	Hypothetical protein [*Escherichia* phage vB_EcoM_Nami]	QXV73352	NCBI	0.00E+00	Uncharacterized protein	A0A2M9X2R4-F1	AlphaFold	4.60E-121
ORF158	tRNA-Arg(tct)	-	-	-	-	-	-	-	-
ORF159	Nuclease	GIY-YIG nuclease family protein [*Salmonella* enterica]	WP_080181680	NCBI	0.00E+00	GIY-YIG domain-containing protein	A0A7X7A1M3-F1	AlphaFold	8.30E-63
ORF160	tRNA-Met(cat)	-	-	-	-	-	-	-	-
ORF161	tRNA-Thr(tga)	-	-	-	-	-	-	-	-
ORF162	tRNA-Ser(tga)	-	-	-	-	-	-	-	-
ORF163	tRNA-Pro(tgg)	-	-	-	-	-	-	-	-
ORF164	tRNA-Gly(tcc)	-	-	-	-	-	-	-	-
ORF165	tRNA-Leu(tcc)	-	-	-	-	-	-	-	-
ORF166	HNH endonuclease	HNH endonuclease [*Escherichia coli*]	EFD8933283	NCBI	0.00E+00	HNHc domain-containing protein	D9RX18-F1	AlphaFold	1.40E-48
ORF167	Hypothetical protein	Hypothetical protein bas38_0257 [*Escherichia* phage AugustSocin]	QXV76457	NCBI	0.00E+00	Zinc finger protein	A0A4Y2PNK4-F1	AlphaFold	0.000078
ORF168	Central straight tail fiber	PHAGE_Shigel_Sf24_NC_042078: central straight tail fiber; phage(gi100275)	PP_00164	PHASTER	9.48E-63	Uncharacterized protein	A0A2M9X3N1-F1	AlphaFold	3.60E-38

ORF	Predicted function	Sequence similarlity	Accession no.	Database	E-value	Predited function through protein structure	Accession no.	Database	E-value
ORF169	Hypothetical protein	Hypothetical protein [*Escherichia* phage VEc20]	QDK04266	NCBI	0.00E+00	Uncharacterized protein	A0A2M9X3G5-F1	AlphaFold	9.10E-59
ORF170	Hypothetical protein	MULTISPECIES hypothetical protein [Bacteria]	WP_015969296	NCBI	1.10E-23	Uncharacterized protein	A0A2M9X3H8-F1	AlphaFold	7.50E-26
ORF171	Putative internal head protein	Putative internal head protein [*Escherichia* phage JLBYU31]	UGO55102	NCBI	0.00E+00	Uncharacterized protein	A0A2M9X3G4-F1	AlphaFold	1.60E-43
ORF172	MazF family toxin-antitoxin system protein	PHAGE_Escher_CF2_NC_041919: MazF family toxin-antitoxin system protein; phage(gi100109)	PP_00168	PHASTER	2.34E-54	Uncharacterized protein	A0A2M9X3I7-F1	AlphaFold	1.70E-47
ORF173	Distal tail protein	PHAGE_Shigel_Sf24_NC_042078: distal tail protein; phage(gi100279	PP_00169	PHASTER	4.46E-109	Acb1; Anti-CBASS, Nuclease, Immune evasion, VIRAL PROTEIN; 1.14A {*Erwinia* phage FBB1}	7T26_A	PDB	1.10E-20
ORF174	Chaperone for tail fiber formation	Chaperone for tail fiber formation [*Escherichia* phage RB3]	YP_009098528	NCBI	4.20E-43	Uncharacterized protein	A0A2M9X3F0-F1	AlphaFold	1.00E-35
ORF175	Deoxynucleoside monophosphate kinase	Deoxynucleoside monophosphate kinase [*Bacillus cereus*]	WP_100771523	NCBI	0.00E+00	Deoxynucleotide monophosphate kinase	A0A2D7GUE1-F1	AlphaFold	7.10E-53
ORF176	Glycoprotein 3	Glycoprotein 3 [*Escherichia* phage T4]	AAA50419	NCBI	0.00E+00	Tail tube protein	5IV5_p	PDB	2.3e-24
ORF177	DNA end protector during packaging	DNA end protector during packaging [*Escherichia* phage ime09]	YP_007004525.1	NCBI	0.00E+00	DNA end protector protein	A0A2M9X3F2-F1	AlphaFold	4.70E-93
ORF178	Head completion	Head completion [*Escherichia* phage ime09]	YP_007004526.1	NCBI	0.00E+00	Head completion protein	A0A2E4WMT0-F1	AlphaFold	1.5e-59
ORF179	Baseplate wedge protein	Baseplate wedge protein 53 [*Escherichia* phage T4]	NP_049756.1	NCBI	0.00E+00	Baseplate wedge subunit	A0A2M9X3F6-F1	AlphaFold	4.50E-72
ORF180	Glycoside hydrolase family protein	Glycoside hydrolase family protein [*Escherichia* phage ime09]	YP_007004528.1	NCBI	0.00E+00	Tail-associated lysozyme	1WTH_A	PDB	1.50E-65
ORF181	Hypothetical protein	Hypothetical protein *Shigella* phage CT01	UDY80545	NCBI	0.00E+00	Uncharacterized protein	A0A2M9X3H7-F1	AlphaFold	1.60E-55
ORF182	PAAR domain-containing protein	PAAR domain-containing protein [*Escherichia* phage T4]	NP_049763.1	NCBI	0.00E+00	PAAR repeat-containing protein	A0A6N7AIV8-F1	AlphaFold	8.50E-42

ORF	Predicted function	Sequence similarlity	Accession no.	Database	E-value	Predited function through protein structure	Accession no.	Database	E-value
ORF183	Baseplate wedge subunit	Baseplate wedge subunit [*Escherichia coli*]	WP_171921381	NCBI	0.00E+00	Baseplate wedge protein	5HX2_E	PDB	5.00E-103
ORF184	Baseplate wedge subunit	Baseplate wedge subunit [*Escherichia* phage UGKSEcP2]	CAH1615457	NCBI	0.00E+00	Baseplate wedge subunit	A0A2M9X3P4-F1	AlphaFold	0.00E+00
ORF185	Baseplate wedge subunit	Baseplate wedge subunit [*Escherichia* phage UGKSEcP2]	CAH1615459	NCBI	0.00E+00	Baseplate wedge subunit	A0A2E4W7V4-F1	AlphaFold	1.90E-111
ORF186	Baseplate wedge subunit	Baseplate wedge tail fiber connector [*Escherichia coli*]	WP_171921396	NCBI	0.00E+00	Baseplate wedge tail fiber connecto	A0A2E4W8M6-F1	AlphaFold	6.80E-87
ORF187	Baseplate wedge subunit and tail pin	Baseplate wedge subunit and tail pin [*Shigella* phage vB_SsoM_113]	CAA7537947	NCBI	0.00E+00	Phage tail protein	A0A2M9X3L1-F1	AlphaFold	3.80E-284
ORF188	Baseplate wedge subunit and tail pin	Baseplate wedge completion tail pin [*Escherichia coli*]	WP_016054180	NCBI	0.00E+00	Phage tail protein	A0A2M9X3E6-F1	AlphaFold	1.00E-80
ORF189	Tail Collar Domain	Tail Collar Domain	UVX66442.1	NCBI	0.00E+00	Short tail fiber protein	5IV5_n	PDB	7.50E-33
ORF190	Fibritin protein	Fibritin protein [*Escherichia* phage vB_EcoM_WL-3]	QOQ37527	NCBI	0.00E+00	Fibritin	2BSG_A	PDB	1.80E-95
ORF191	Neck protein	Neck protein [*Escherichia coli*]	HBN5609832	NCBI	0.00E+00	Uncharacterized protein	A0A350IB31-F1	AlphaFold	2.40E-98
ORF192	Head closure Hc2	Head closure Hc2 [*Escherichia* phage UGKSEcP2]	CAH1615473	NCBI	0.00E+00	Neck protein	A0A2M9X3F1-F1	AlphaFold	1.30E-87
ORF193	Tail sheath stabilizer and completion protein	Tail sheath stabilizer and completion protein [*Enterobacteria* phage T6]	AXN58197	NCBI	0.00E+00	Tail connector protein	4HUD_B	PDB	1.60E-76
ORF194	Small terminase protein	Small terminase protein [*Escherichia* phage T4]	WP_015969323	NCBI	0.00E+00	Terminase DNA packaging enzyme small subunit	3TXS_B	PDB	6.50E-35
ORF195	Terminase family protein	Terminase family protein [*Escherichia* phage slur02]	YP_009210351.1	NCBI	0.00E+00	DNA packaging protein Gp17; large terminase	3CPE_A	PDB	3.00E-64
ORF196	Phage tail protein	Phage tail protein [Shigella flexneri]	EFW4639777	NCBI	0.00E+00	Tail sheath protein Gp18; bacteriophage T4	3J2M_V	PDB	1.30E-73

ORF	Predicted function	Sequence similarlity	Accession no.	Database	E-value	Predited function through protein structure	Accession no.	Database	E-value
ORF197	Tail protein	Tail protein [*Shigella* phage Shfl2]	YP_004415061	NCBI	0.00E+00	Tail tube protein	5IV5_p	PDB	4.20E-32
ORF198	Portal protein	Portal protein [Escherichia phage vB_EcoM_IME537]	YP_010070937	NCBI	0.00E+00	Portal protein	6UZC_P	PDB	7.40E-84
ORF199	Putative prohead core protein	Putative prohead core protein [*Escherichia* phage vB_EcoM_Kelasse]	QXN69708	NCBI	2.50E-25	Prohead core protein	A0A2M9X3E8-F1	AlphaFold	1.50E-20
ORF200	Prohead core protein	Prohead core protein [*Shigella* phage SHFML-11]	YP_009277543	NCBI	0.00E+00	Prohead core protein	A0A2M9X3D5-F1	AlphaFold	4.90E-63
ORF201	Prohead core scaffolding protein and protease	Prohead core scaffolding protein and protease [*Escherichia coli*]	HBK9476213	NCBI	0.00E+00	Prohead core protein	5JBL_E	PDB	1.70E-47
ORF202	Prohead core protein	Prohead core protein [*Escherichia coli*]	EFG3114296	NCBI	0.00E+00	T4 prohead core scaffold protein	A0A2E9U1V5-F1	AlphaFold	2.00E-82
ORF203	Major capsid protein	Major capsid protein [*Shigella* phage ESh30]	URY14669.1	NCBI	0.00E+00	Phage capsid protein	A0A2M9X3C2-F1	AlphaFold	3.80E-147
ORF204	Capsid vertex protein	Capsid vertex protein [*Escherichia coli*]	WP_247010967	NCBI	0.00E+00	Head vertex protein	1YUE_A	PDB	4.30E-90
ORF205	RNA ligase	RNA ligase Rnl2 family [*Escherichia* phage vB_EcoM_OE5505]	YP_010072465	NCBI	0.00E+00	RNA Ligase	1S68_A	PDB	1.90E-32
ORF206	Hypothetical protein	Hypothetical protein Shfl2p178 [*Shigella* phage Shfl]	YP_004415070.1	NCBI	0.00E+00	Uncharacterized protein	A0A2M9X3J1-F1	AlphaFold	2.10E-53
ORF207	Hypothetical protein	Phage protein [Yersinia phage fPS-65]	YP_010091483	NCBI	7.40E-35	Uncharacterized protein	A0A2M9X3C6-F1	AlphaFold	1.70E-23
ORF208	Putative head outer capsid protein	Putative head outer capsid protein [*Escherichia* phage U115]	UAV89140	NCBI	0.00E+00	Highly immunogenic outer capsid protein	5VF3_Z	PDB	3.30E-15
ORF209	Inhibitor of prohead protease	Inhibitor of prohead protease [*Salmonella* phage SG1]	YP_010075074	NCBI	0.00E+00	Preprotein translocase subunit Tim44	A0A2M9X3C3-F1	AlphaFold	5.30E-89
ORF210	DNA helicase	DNA helicase [*Yersinia* phage vB_YepM_ZN18]	QIG57235.1	NCBI	0.00E+00	ATP-dependent DNA helicase uvsW	2OCA_A	PDB	1.20E-61

ORF	Predicted function	Sequence similarlity	Accession no.	Database	E-value	Predited function through protein structure	Accession no.	Database	E-value
ORF211	Homing endonuclease	Homing endonuclease [*Shigella* phage vB_SsoM_113]	CAA7537897	NCBI	0.00E+00	HNH endonuclease	A0A2M9X372-F1	AlphaFold	3.70E-54
ORF212	DNA helicase	DNA helicase [*Escherichia* phage UGKSEcP2]	CAH1615509	NCBI	2.80E-45	ATP-dependent DNA helicase uvsW	2JPN_A	PDB	4.10E-45
ORF213	Head morphogenesis protein	PHAGE_Escher_CF2_NC_041919: head morphogenesis protein; phage(gi100068)	PP_00209	PHASTER	3.06E-33	DUF2685 domain-containing protein	WP_015969346.1	BlastX	5.00E-20
ORF214	Hypothetical protein	Hypothetical protein FDJ02_gp220 [*Shigella* phage Sf21]	YP_009618992	NCBI	0.00E+00	Uncharacterized protein	A0A2M9X3D7-F1	AlphaFold	5.60E-41
ORF215	Recombination mediator protein UvsY	Recombination mediator protein UvsY [*Escherichia coli*]	WP_247010961	NCBI	0.00E+00	Recombination protein uvsY	4ZWS_B	PDB	1.00E-45
ORF216	Baseplate wedge subunit	Baseplate wedge subunit [*Shigella* phage ESh36]	URY16176	NCBI	0.00E+00	Baseplate wedge protein	5IW9_B	PDB	6.50E-24
ORF217	Base plate protein	T4 bacteriophage base plate protein [*Escherichia* phage slur07]	YP_009197268.1	NCBI	0.00E+00	Baseplate protein	A0A2D6LTE1-F1	AlphaFold	7.50E-79
ORF218	Base plate protein	Baseplate protein [*Escherichia* phage MLP2]	UEN68704.1	NCBI	0.00E+00	Baseplate hub assembly protein	A0A2M9X3C9-F1	AlphaFold	7.10E-105
ORF219	Base plate protein	Base plate hub subunit [*Escherichia* phage HY03]	YP_009284054.1	NCBI	0.00E+00	Baseplate hub subunit	A0A2M9X3B3-F1	AlphaFold	1.30E-194
ORF220	Base plate protein	Putative baseplate distal hub subunit [*Escherichia* phage HY01]	YP_009148631	NCBI	0.00E+00	Uncharacterized protein	A0A2M9X3D3-F1	AlphaFold	1.80E-73
ORF221	Baseplate hub subunit and tail length	Baseplate hub subunit and tail length [*Escherichia* phage UGKSEcP2]	CAH1615527.1	NCBI	0.00E+00	Uncharacterized protein	A0A2M9X3C4-F1	AlphaFold	3.30E-211
ORF222	Base plate protein	Baseplate tail-tube junction protein [*Escherichia coli*]	MBJ0221106	NCBI	0.00E+00	Baseplate tail-tube protein	5IV5_ID	PDB	3.60E-86
ORF223	Tail assembly protein	Tail assembly protein [*Shigella* phage ESh16]	URY11480	NCBI	0.00E+00	Baseplate tail-tube protein	5IV5_DJ	PDB	1.60E-65
ORF224	Tail length tape-measure protein	PHAGE_Escher_CF2_NC_041919: tail length tape-measure protein; phage(gi100057)	PP_00220	PHASTER	4.04E-62	Uncharacterized protein	A0A2M9X3C7-F1	AlphaFold	9.60E-36

ORF	Predicted function	Sequence similarlity	Accession no.	Database	E-value	Predited function through protein structure	Accession no.	Database	E-value
ORF225	RNA polymerase-ADP-ribosyltransferase	RNA polymerase-ADP-ribosyltransferase [*Escherichia* phage EP01]	UIU46944	NCBI	0.00E+00	ADP-ribosyltransferase	A0A2M9X3A2-F1	AlphaFold	5.00E-241
ORF226	ADP-ribosyltransferase	ADP-ribosyltransferase [*Escherichia* phage EC121]	YP_010067878	NCBI	0.00E+00	ADP-ribosyltransferase	A0A2M9X3A2-F1	AlphaFold	2.60E-243
ORF227	Putative single-stranded DNA binding protein	PHAGE_Shigel_Sf24_NC_042078: putative single-stranded DNA binding protein; phage(gi100050)	PP_00223	PHASTER	1.40E-38	Uncharacterized protein	A0A2M9X3H3-F1	AlphaFold	1.10E-32
ORF228	DNA ligase	DNA ligase [*Shigella* phage vB_SsoM_113]	CAA7537862	NCBI	0.00E+00	DNA ligase	6DT1_A	PDB	5.30E-70
ORF229	Hypothetical protein	DUF3045 domain-containing protein [*Bacillus cereus*]	WP_074146535	NCBI	0.00E+00	Uncharacterized protein	A0A2M9X3B9-F1	AlphaFold	4.50E-36
ORF230	Hypothetical protein	Hypothetical protein FDJ02_gp204 [*Shigella* phage Sf21]	YP_009619008	NCBI	0.00E+00	Probable phosphoserine phosphatase	2OM6_B	PDB	2.60E-17
ORF231	Hypothetical protein	Hypothetical protein UGKSECP2_00220 [*Escherichia* phage UGKSEcP2]	CAH1615547	NCBI	0.00E+00	Hypothetical protein	2B3W_A	PDB	2.30E-29
ORF232	Putative baseplate hub	Putative baseplate hub [*Escherichia* phage UPEC07]	QUL77534	NCBI	1.40E-31	Uncharacterized protein	A0A2M9X3B4-F1	AlphaFold	3.80E-30
ORF233	Hypothetical protein	Hypothetical protein UGKSECP2_00222 [*Escherichia* phage UGKSEcP2]	CAH1615551	NCBI	0.00E+00	Uncharacterized protein	A0A2M9X3J6-F1	AlphaFold	1.10E-55
ORF234	Putative baseplate hub	Putative base plate hub subunit [*Escherichia* phage HY03]	YP_009284255	NCBI	0.00E+00	Uncharacterized protein	A0A2M9X3I9-F1	AlphaFold	2.70E-51
ORF235	Putative transcriptional regulator	PHAGE_Escher_CF2_NC_041919: putative transcriptional regulator; phage(gi100045)	PP_00231	PHASTER	7.86E-76	Uncharacterized protein	A0A2M9X3B8-F1	AlphaFold	3.90E-39
ORF236	Hypothetical protein	Hypothetical protein KMC07_gp201 [*Escherichia* phage vB_EcoM_G8]	YP_010069500	NCBI	1.50E-21	Uncharacterized protein	A0A085LM69-F1	AlphaFold	6.00E-08
ORF237	rIII lysis inhibition accessory protein	rIII lysis inhibition accessory protein rapid lysis phenotype [*Escherichia* phage T4]	NP_049824.1	NCBI	0.00E+00	Uncharacterized protein	A0A2M9X393-F1	AlphaFold	8.30E-42
ORF238	Co-chaperone GroES family protein	Co-chaperone GroES family protein [*Escherichia* phage T4]	NP_049825.1	NCBI	0.00E+00	CO-CHAPERONIN	1G31_D	PDB	5.40E-28

ORF	Predicted function	Sequence similarlity	Accession no.	Database	E-value	Predited function through protein structure	Accession no.	Database	E-value
ORF239	Hypothetical protein	Hypothetical protein UGKSECP2_00228 [*Escherichia* phage UGKSEcP2]	CAH1615563	NCBI	0.00E+00	Uncharacterized protein	A0A2M9X392-F1	AlphaFold	1.50E-35
ORF240	Tail fibers protein	Tail fibers protein [*Enterobacteria* phage GiZh]	YP_010066166.1	NCBI	0.00E+00	Uncharacterized protein	A0A2M9X3A9-F1	AlphaFold	4.70E-39
ORF241	dCMP deaminase	dCMP deaminase [*Enterobacteria* phage RB51]	YP_002854168.1	NCBI	0.00E+00	DEOXYCYTIDYLATE DEAMINASE	1VQ2_A	PDB	6.10E-33
ORF242	Thymidylate synthase	PHAGE_Escher_slur04_NC_042130: thymidylate synthase; phage(gi100195)	PP_00238	PHASTER	9.90E-77	Uncharacterized protein	A0A2M9X3B0-F1	AlphaFold	1.20E-42
ORF243	Hypothetical protein	Hypothetical protein [*Escherichia coli*]	WP_016039623	NCBI	0.00E+00	Uncharacterized protein	A0A2M9X3A7-F1	AlphaFold	5.70E-31
ORF244	Hypothetical protein	Hypothetical protein UGKSECP2_00233 [*Escherichia* phage UGKSEcP2]	CAH1615573	NCBI	0.00E+00	Uncharacterized protein	A0A2M9X390-F1	AlphaFold	2.20E-114
ORF245	Hypothetical protein	Hypothetical protein Shfl2p219 [*Shigella* phage Shfl2]	YP_004415110.1	NCBI	0.00E+00	Uncharacterized protein	A0A2M9X3H9-F1	AlphaFold	2.20E-35
ORF246	Hypothetical protein	Hypothetical protein [*Escherichia coli*]	WP_015995822	NCBI	4.20E-39	Uncharacterized protein	A0A2M9X3A8-F1	AlphaFold	6.70E-33
ORF247	Hypothetical protein	Hypothetical protein KMC13_gp059 [*Escherichia* phage vB_EcoM_IME537]	YP_010070987	NCBI	2.80E-40	Uncharacterized protein	A0A2M9X380-F1	AlphaFold	4.20E-35
ORF248	3'-phosphatase 5'-polynucleotide kinase	3'-phosphatase 5'-polynucleotide kinase [*Yersinia* phage PYps32T]	QNJ50047.1	NCBI	0.00E+00	POLYNUCLEOTIDE KINASE	1LTQ_A	PDB	9.80E-37
ORF249	DNA helicase	PHAGE_Shigel_SHFML_26_NC_031011: DNA helicase; phage(gi100044)	PP_00245	PHASTER	4.01E-70	Uncharacterized protein	A0A2M9X378-F1	AlphaFold	4.90E-45
ORF250	Flap endonuclease	PHAGE_Shigel_Sf22_NC_042039: flap endonuclease; phage(gi100131)	PP_00246	PHASTER	7.93E-51	Uncharacterized protein	A0A2M9X3A0-F1	AlphaFold	5.40E-31
ORF251	Phage outer membrane lipoprotein Rz1	Phage outer membrane lipoprotein Rz1 [*Escherichia* phage ime09]	YP_007004598.1	NCBI	0.00E+00	Uncharacterized protein	A0A2M9X382-F1	AlphaFold	1.30E-37
ORF252	Putative spanin inner membrane subunit	Putative spanin inner membrane subunit [*Escherichia* phage 132]	QWY90593	NCBI	1.40E-45	Uncharacterized protein	A0A2M9X399-F1	AlphaFold	3.50E-38

ORF	Predicted function	Sequence similarlity	Accession no.	Database	E-value	Predited function through protein structure	Accession no.	Database	E-value
ORF253	Inhibitor of host transcription	Inhibitor of host transcription [*Escherichia* phage BF15]	QXN75925	NCBI	0.00E+00	Uncharacterized protein	A0A2M9X397-F1	AlphaFold	3.50E-70
ORF254	T4 RnlA family RNA ligase	T4 RnlA family RNA ligase [*Salmonella* phage pSe_SNUABM_01]	YP_010075493	NCBI	0.00E+00	T4 RNA ligase	5TT6_A	PDB	5.40E-61
ORF255	Endonuclease	Endonuclease pEscherichia phage vB_EcoM_Nami]	QXV73394	NCBI	0.00E+00	ENDONUCLEASE II	2WSH_C	PDB	5.80E-19
ORF256	Ribonucleotide reductase of class Ia aerobic beta subunit	Ribonucleotide reductase of class Ia aerobic beta subunit [*Salmonella* phage GRNsp7]	USW07498	NCBI	0.00E+00	Ribonucleotide reductase R2	1MXR_A	PDB	1.10E-53
ORF257	Putative homing endonuclease	Putative homing endonuclease [*Enterobacteria* phage RB15]	ABA03242	NCBI	0.00E+00	HNH endonuclease	A0A843FSR4-F1	AlphaFold	8.00E-45
ORF258	Ribonucleoside-diphosphate reductase subunit alpha	Ribonucleoside-diphosphate reductase subunit alpha [*Enterobacteria* phage vB_EcoM_IME340]	YP_010066426	NCBI	0.00E+00	RIBONUCLEOSIDE-DIPHOSPHATE REDUCTASE 1 SUBUNIT ALPHA	2XAP_B	PDB	1.50E-114
ORF259	Hypothetical protein	DUF4326 domain-containing protein [*Bacillus cereus*]	WP_016039635	NCBI	0.00E+00	DNA-directed RNA polymerase	A0A0U2B1N9-F1	AlphaFold	1.60E-35
ORF260	Tail length tape-measure protein	PHAGE_Shigel_SHFML_26_NC_031011: tail length tape-measure protein; phage(gi100057)	PP_00256	PHASTER	4.46E-58	Uncharacterized protein	A0A2M9X369-F1	AlphaFold	1.20E-41
ORF261	Thymidylate synthase	Thymidylate synthase [*Yersinia* phage vB_YepM_ZN18]	YP_010077467	NCBI	0.00E+00	THYMIDYLATE SYNTHASE	1TIS_A	PDB	4.00E-63
ORF262	Hypothetical protein	Hypothetical protein SP1_0042 [*Escherichia* phage vB_EcoM_SP1]	QLF80895	NCBI	0.00E+00	Hypothetical protein	QLF80895.1	BlastX	3.00E-80
ORF263	Dihydrofolate reductase	Dihydrofolate reductase [Escherichia phage RB3]	YP_009098619	NCBI	0.00E+00	DIHYDROFOLATE REDUCTASE	1JUV_A	PDB	4.50E-30
ORF264	Pore-forming tail tip protein	PHAGE_Shigel_SHFML_11_NC_030953: pore-forming tail tip protein; phage(gi100139)	PP_00260	PHASTER	2.78E-51	Uncharacterized protein	A0A2M9X3F9-F1	AlphaFold	1.60E-53
ORF265	DNA adenine methyltransferase	PHAGE_Escher_slur03_NC_042129: DNA adenine methyltransferase; phage(gi100041)	PP_00261	PHASTER	1.51E-74	Uncharacterized protein	A0A6L6S0Y4-F1	AlphaFold	0.000044
ORF266	Hypothetical protein	DUF5417 domain-containing protein [*Escherichia* phage vB_EcoM-UFV13]	YP_009290500	NCBI	0.00E+00	Frd.1 domain protein	A0A1W6C1B6-F1	AlphaFold	2.10E-12

ORF	Predicted function	Sequence similarlity	Accession no.	Database	E-value	Predited function through protein structure	Accession no.	Database	E-value
ORF267	Hypothetical protein	Hypothetical protein [*Shigella flexneri*]	EFW4203867	NCBI	0.00E+00	Uncharacterized protein	A0A2M9X362-F1	AlphaFold	1.40E-46
ORF268	Putative single stranded DNA-binding protein	Putative single stranded DNA-binding protein [*Escherichia* phage JLBYU24]	UGO55585	NCBI	4.20E-45	Uncharacterized protein	A0A2M9X3D2-F1	AlphaFold	1.50E-40
ORF269	ssDNA binding protein	ssDNA binding protein [*Escherichia* phage vB_EcoM_SP1]	QLF80803	NCBI	0.00E+00	Single-stranded DNA-binding protein	A0A2E1I8U5-F1	AlphaFold	6.80E-105
ORF270	Loader of DNA helicase and ssDNA binding	Loader of DNA helicase and ssDNA binding [*Enterobacteria* phage RB9]	AIT74061.1	NCBI	0.00E+00	BPT4 GENE 59 HELICASE ASSEMBLY PROTEIN	1C1K_A	PDB	2.40E-76
ORF271	Late promoter transcription accessory protein	Late promoter transcription accessory protein [*Escherichia* phage T4]	NP_049857.1	NCBI	0.00E+00	RNA polymerase-associated protein	3TBI_A	PDB	5.70E-42
ORF272	Double-stranded DNA binding protein	Double-stranded DNA binding protein [*Escherichia* phage EP01]	UIU46989.1	NCBI	0.00E+00	Double-stranded DNA-binding protein	A0A2M9X383-F1	AlphaFold	2.00E-38
ORF273	Endolysin	PHAGE_Escher_slur03_NC_042129: endolysin; phage(gi100033)	PP_00269	PHASTER	0.00E+00	Ribonuclease H	3H7I_A	PDB	9.10E-49
ORF274	Tail protein	Tail protein [*Escherichia* phage EcNP1]	YP_010068151	NCBI	0.00E+00	Long-tail fiber proximal subunit	5NXF_C	PDB	2.20E-91
ORF275	Long tail fiber	Long tail fiber [*Escherichia* phage vB_EcoM_Shinka]	QXV73050	NCBI	0.00E+00	ILEI domain-containing protein	A0A2M9X3E9-F1	AlphaFold	4.00E-127
ORF276	Tail fiber protein	Tail fiber protein [*Escherichia* phage YUEEL01]	YP_010074966	NCBI	0.00E+00	Phage T4 tail fiber	A0A1C3HHN2-F1	AlphaFold	3.50E-29
